# Opportunity costs of carbon sequestration in a forest concession in central Africa

**DOI:** 10.1186/s13021-014-0004-3

**Published:** 2014-07-03

**Authors:** Michel Ndjondo, Sylvie Gourlet-Fleury, Raphaël J Manlay, Nestor Laurier Engone Obiang, Alfred Ngomanda, Claudia Romero, Florian Claeys, Nicolas Picard

**Affiliations:** 1 Institut de Recherches en Écologie Tropicale, Libreville, BP 13354 Gabon; 2 Centre de Coopération Internationale en Recherche Agronomique pour le Développement, BSEF research unit, Campus international de Baillarguet, Montpellier, 34398 Cedex 5 France; 3 AgroParisTech, UMR Eco&Sols, Montpellier SupAgro, Montpellier, 34060 Cedex 2 France; 4 Department of Biology, University of Florida, P.O Box 118525, Gainesville, 32611 FL USA; 5 CIRAD, Yaoundé, BP 2572 Cameroon; 6 Present address: Ministère des Eaux et Forêts, Libreville, Gabon

**Keywords:** Break-even price, Carbon credit, Forest degradation, Forest management, REDD+, Tropical forest, Diameter cutting limit, Felling cycle, Forest dynamics

## Abstract

**Background:**

A large proportion of the tropical rain forests of central Africa undergo periodic selective logging for timber harvesting. The REDD+ mechanism could promote less intensive logging if revenue from the additional carbon stored in the forest compensates financially for the reduced timber yield.

**Results:**

Carbon stocks, and timber yields, and their associated values, were predicted at the scale of a forest concession in Gabon over a project scenario of 40 yr with reduced logging intensity. Considering that the timber contribution margin (i.e. the selling price of timber minus its production costs) varies between 10 and US$40 m ^−3^, the minimum price of carbon that enables carbon revenues to compensate forgone timber benefits ranges between US$4.4 and US$25.9/tCO _2_ depending on the management scenario implemented.

**Conclusions:**

Where multiple suppliers of emission reductions compete in a REDD+ carbon market, tropical timber companies are likely to change their management practices only if very favourable conditions are met, namely if the timber contribution margin remains low enough and if alternative management practices and associated incentives are appropriately chosen.

**Electronic supplementary material:**

The online version of this article (doi:10.1186/s13021-014-0004-3) contains supplementary material, which is available to authorized users.

## Background

Tropical forests of central Africa provide both global and regional ecosystem services [1]. They provide provisioning services to the people that live within and around the forests. In several countries of central Africa, timber is the second most important sector of the economy after oil [2]. Non-timber forest products extracted from forests, including game animals, are important to local populations [3]. Forests provide regulating services such as climate change mitigation by carbon storage from the atmosphere, with a likely increase in forest productivity due to the increase in atmospheric CO _2_[4],[5]. Forests also provide cultural services. The Lopé National Park in Gabon, for instance, that has been classified as a cultural and natural property of the UNESCO World Heritage, is part of a network of forest national parks that besides delivering provisioning and regulating services, aims at promoting ecotourism and wildlife observation [6].

The multiple possible uses of tropical forests in central Africa imply the existence of trade-offs between different forest users and ecosystem services [7]-[9]. With the increasing interest in reduced emissions from deforestation and forest degradation, conservation, sustainable management of forests, and enhancement of forest carbon stocks (REDD+; [10]), a salient trade-off is arising between timber production and carbon sequestration. Many carbon cost-benefit studies have either been devoted to plantations (i.e., afforestation, and reforestation; [8],[11],[12]), or to comparisons between timber production and forest conversion to crops as alternatives to forest conservation [13]-[16]. Less attention has been given so far to changes in natural forest management practices, in particular to the outcomes of variations of timber harvesting intensity and associated policy scenarios (but see [17]). Phat et al. [18] developed management scenarios and estimated carbon offset benefits from both carbon accrued through regeneration and growth of trees in residual stands. The authors calculated the minimum price of carbon needed to offset the costs of reduced impact logging (RIL) relative to conventional harvest practices but did not consider other costs related to the implementation of improved practices, which can be substantial (e.g., foregone benefits of reduced timber harvests) and the financial consequences of management decisions. Related work by Sasaki et al. [19] calculated the annual equivalent value in one hectare for six alternative land use transitions in Cambodia considering total costs (i.e., costs to the government, to the logging companies, and to REDD+ implementers) and rents (i.e., derived from timber sales, taxes and royalties, and potential carbon sales) for a range of stakeholders. The authors then compared changes in the annual equivalent value of these alternative land uses under different carbon prices and discount rates. Their results found highest values for both the business as usual and the timber-REDD+ scenarios.

Few carbon cost-benefit studies have been conducted in Africa as compared to other parts of the world. Chisholm [7] investigated the trade-off between water and carbon in timber plantations in South Africa; Bellassen and Gitz [20] and Merger et al. [21] investigated the trade-off between shifting cultivation and forest conservation in Cameroon and Tanzania, respectively; in their study of the potential of improved management of natural forests of central Africa to mitigate climate change, Durrieu et al. [22] restricted their investigation to the quantitative characterization of the carbon balance. Thus, whereas a large portion of central African forests is covered with natural productive forests under concession and management plans, we are not aware of any carbon cost-benefit studies dealing with the analysis of policy scenarios that consider changes in management practices in these forests [9].

The management of natural forests under concession in central Africa has specific features that could make them eligible to REDD+ [9] along different lines of the already approved REDD+ initiative in two Peruvian logging concessions [23]. In this study, we considered two management practices that could be modified to improve carbon storage to the detriment of timber production over the time horizon of a felling cycle. The first one is the lengthening of the felling cycle [11]. The second one is the raising of the minimum diameter cutting limits. This study addresses two main questions in the particular case of a forest concession in Gabon: (1) what is the carbon accretion potential of these two management options; and, (2) what is the break-even price of carbon credits that would make each of these two policy options cost-neutral to the concessions holder? In contrast to prior research [18],[19], our approach captures the opportunity costs of alternative management scenarios through time, including the foregone benefits linked to specific policy options. Our work intends to construct a break-even value for the price of carbon that would be needed to cover the opportunity costs of plausible policy scenarios. We highlight that even if we recognize the attributes that RIL-based practices can have for carbon and other forest ecosystem services, we consider that an attempt to model changes that include RIL implementation and other improved forest management practices would be far beyond what could be realistically aimed in the short term in Gabonese forests.

The opportunity costs of carbon accrediting management options are assessed from the viewpoint of the forest concessionaire (i.e., the party with the right to log), at the spatial scale of a forest concession, and at the time horizon of a REDD+ project (i.e. 40 yr). This time horizon seems like a good compromise between the timing of forest ecological dynamics and the limited attractiveness of long-term economic planning due to politico-economic uncertainty. Because the characteristics of future REDD+ projects are not yet clear, we used the methods defined by the Verified Carbon Standard (VCS) for improved forest management projects [24],[25] to calculate the break-even carbon price and associated risk assessment for lengthened cutting cycles and increased minimum cutting diameters. Whenever relevant, we also complied with the technical standards defined by the forest legislation in Gabon. In particular, management parameters (including tree species growth rates, logging damage, etc.) were taken from the management plan of the forest concession. Computations were based on real tree population data from the forest concession in Gabon, with a virtual implementation of the REDD+ project in this concession following the VCS methodology. All future forest dynamics (including during the virtual REDD+ project) were predicted using a classical model of forest dynamics based on transition matrices [26].

## Results

### Timber and carbon dynamics

**Figure 1:**
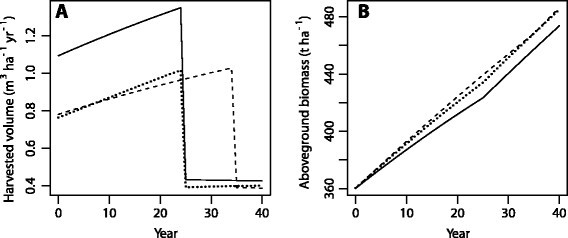
**Temporal forecasts in the Haut-Abanga forest concession, Gabon.** Harvested wood volume (panel **A**) and aboveground biomass (panel **B**) are forecast according to three management scenarios: solid line is the reference scenario (*T*^ref^, $d_{s}^{\text{ref}}$); dashed line is an alternative scenario with a longer felling cycle *T*=*T*^ref^+10 yr; and dotted line is an alternative scenario with higher cutting limit diameters *d*_*s*_=*d* *s* ref+10 cm.

The initial harvestable timber volume in the forest concession in Gabon was 27.4 m^3^ ha^−1^ (logging intensity: 90% of available number of commercial trees) or 30.4 m^3^ ha^−1^ (logging intensity: 100%). Harvested volumes varied between 0.39 and 1.35 m^3^ ha^−1^ yr^−1^ depending on the year and on the management scenario (Figure 1A). The baseline scenario (i.e., business-as-usual), that corresponds to the current management practices, yielded the highest harvested volumes until the 25th year (Figure 1A, solid line), at which time the whole concession had been logged once. Starting in year 26, the concession underwent a second cut but harvested volumes were then much lower from the first cut (by 65% on average). This result means that the first cut took advantage of the initial stock, which was then depleted for the subsequent rotations (i.e. primary forest premium, as coined by [27]). When the diameter cutting limits were raised, harvested volumes decreased (by 24% on average for +10 cm; Figure 1A, dotted line). When the felling cycle was lengthened, harvested volumes also decreased (by 9% on average for +10 yr) when compared to the baseline scenario but it remained longer at its highest level (Figure 1A, dashed line).

**Figure 2:**
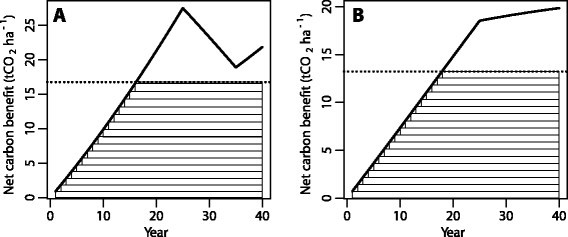
**Net carbon benefit and issuable carbon credits in each year in the Haut-Abanga concession.** The net carbon benefit (thick solid lines) and the issuable carbon credits (bars) are relative to a reference management scenario (*T*^ref^, $d_{s}^{\text{ref}}$). The dotted line is the long-term average of net carbon benefits and the horizontal bars represent the net carbon benefits that area issued each year. **A**. Alternative scenario with a longer rotation *T*=*T*^ref^+10 yr. **B**. Alternative scenario with higher diameter cutting limits *d*_*s*_=*d* *s* ref+10 cm.

The initial aboveground biomass in the concession was 360.3 t ha^−1^, and increased up to 473.7−486.4 t ha^−1^ depending on the management scenario (Figure 1B). On a concession level, biomass increased despite logging because of selectiveness of timber harvesting. Biomass increments were higher with any alternative than with the baseline scenario (Figure 2). However the net carbon benefit did not necessarily accumulate over time (see Figure 2A, for instance, where the net benefit decreases from year 25 to year 35).

To illustrate how carbon credits were issued, consider for example the project scenario with a longer rotation (+10 yr, Figure 2A). In year 1, the net carbon benefit was 0.92 tCO_2_ ha^−1^, and so 0.92 carbon credits were issuable. In year 2, the net carbon benefit increased by an additional 0.93 tCO_2_ ha^−1^ that were issuable as carbon credits. This lasted till year 17 when the accumulated number of issuable carbon credits reached the long-term average of net carbon benefit. No carbon credit was issuable after year 17. This particular calendar of credits issuance follows from the VCS methodology [24] and enables carbon revenues to be quickly obtained after the start of the project. Other methodologies to issue carbon credits have been proposed in the context of the Clean Development Mechanism [12] and would distribute carbon revenues more evenly across the lifetime of the project. All issuable credits summed up to 16.73 tCO_2_ ha^−1^ during the 40 yr project lifetime but due to the buffer for the non-permanence risk, 13.0 carbon credits would actually be issued.

### Opportunity cost

The break-even price of carbon sequestration (i.e. the minimum price for a ton of CO_2_ which a forest company would have to receive for the carbon revenues of project scenario to compensate the financial timber losses relative to the baseline scenario) depends on the contribution margins of the sold timber. As long as the same contribution margin *π*_*s*_ is used for all commercial species, the break-even carbon price $\pi_{C}^{*}$ is proportional to this common value. Hence, we do not need to compute the value of $\pi_{C}^{*}$ for all values of *π*_*s*_: if $\pi_{\text{ref}}^{*}$ is the break-even carbon price for a reference value of the contribution margin of for example, US$25 m ^−3^, then the break-even carbon price for a contribution margin of *π*_*s*_ (in US$ m ^−3^) is $\pi_{s}/25\times \pi_{\text{ref}}^{*}$. Therefore, we report the break-even carbon price only for the median *π*_*s*_= US$25 m ^−3^ (Table 1).

**Table 1:** **Opportunity cost of carbon sequestration for different alternative management scenarios in the Haut-Abanga concession**

Scenario	***Δ***Volume	∑Credits	***Δ***PVT_T_	$\pi_{C}^{*}$
	(m^***3***^ha^***−1***^)	(tCO_***2***_ha^***−1***^)	(US$ ha^***−1***^)	(US$/ tCO_***2***_)
Lengthened rotation (+5 yr)	1.8	7.1	34.2	11.0
Lengthened rotation (+10 yr)	3.3	13.0	60.5	11.1
Lengthened rotation (+15 yr)	4.7	18.2	81.2	11.1
Raised cutting limits (+10 cm)	8.9	10.3	65.6	15.8
Raised cutting limits (+20 cm)	17.5	19.5	124.2	15.9
Raised cutting limits (+30 cm)	24.5	26.3	166.2	16.1
Raised cutting limits (+40 cm)	29.5	30.7	193.6	16.2

The break-even price of carbon sequestration $\pi_{C}^{\text{*}}$ is computed for a contribution margin of US$25 m ^−3^ for all commercial species. ∑Credits is the sum of carbon credits. *Δ* Volume is the total reduction in harvested wood volume. *Δ* PVT_T_ is the loss in the net present value of timber.

For a contribution margin of US$25 m^−3^, the break-even price of carbon was US$11.0–16.2/tCO _2_ depending on the project scenario (Table 1). When lengthening the rotation from an additional 5 yr to an additional 10 or 15 yr, the gain in carbon credits for each type of credit increased but so did the loss in the net present value of timber so that, eventually, the break-even price for both was about US$11/ tCO_2_ for a contribution margin of US$25 m^−3^. Similarly, raising the diameter cutting limit from an additional 10 cm to 20, 30 or 40 cm did not much affect the opportunity cost of carbon sequestration. The break-even price was higher when the diameter cutting limit was increased by 10 cm than when the cutting cycle was lengthened by 10 yr (Table 1).

**Figure 3:**
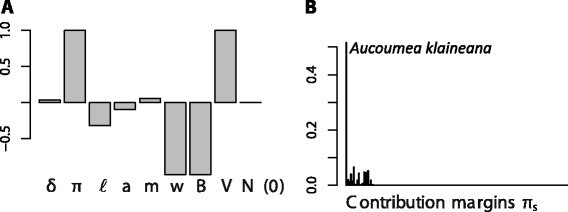
**Results of sensitivity analyses to determine break-even price of carbon.** **A**. Elasticities of the break-even price $\pi_{C}^{*}$ to *δ*= discount rate, ***π***= vector of specific contribution margins, *ℓ*= logging damage, **a**= vector of specific growth rates, **m**= vector of specific mortality rates, **w**= vector of specific wood densities, **B**= vector of class-dependent biomasses, **V**= matrix of species- and class-dependent volumes, and **N**(0)= matrix of initial numbers of trees per species and diameter class. **B**. Elasticities of $\pi_{C}^{*}$ to the species-specific contribution margin *π*_*s*_. Total number of bars is 313, and *Aucoumea klaineana* corresponds to the first bar.

The VCS [25] recommends to account for uncertainties in the estimates of carbon emissions. Hence, a sensitivity analysis was conducted to assess how the break-even price of CO_2_ varies with ecological and economic parameters. The elasticity of the break-even price of carbon to a parameter of the model gives the relative change of the break-even price that is brought by a relative change of this parameter. It quantifies how uncertainty on the parameter value propagates to the estimate of the break-even price. To save space, we present the results of the sensitivity analysis only for the project scenario with a longer rotation *T*^ref^+10 yr. As the break-even price of carbon is directly proportional to the contribution margin of wood, the elasticity of $\pi_{C}^{*}$ to all joined *π*_*s*_ was 1 (Figure 3A). However, as expected, not all species’ prices affected the break-even price of carbon in the same way, with the price of *Aucoumea klaineana* (the most important commercial species) alone being responsible for half the variation of the break-even price (Figure 3B), while the other commercial species were jointly responsible for the other half of the variation of $\pi_{C}^{*}$. The elasticity of the break-even price to the discount rate was low (4%) because most of the credits were issued in the first years of the project.

The elasticity of $\pi_{C}^{*}$ to all joined wood densities was 1 (Figure 3A), evidencing a proportionality dependence of the break-even carbon price on this parameter. The same held true for the biomass for each size-class (for a hypothetical reference species with a wood density of 1 g cm^−3^) and for the volume for each size-class and species (Figure 3A). In comparison, the break-even carbon price was much less sensitive to logging damage, species-specific growth rates and mortality rates, and to the initial tree densities by species and diameter class (Figure 3A and Additional file 1).

## Discussion

The opportunity cost of carbon sequestration in a forest concession in central Africa was assessed for different management scenarios, and the corresponding break-even prices of carbon were estimated. Beyond the specific value of the opportunity cost of carbon calculated for each scenario, the underlying model seems useful. Tropical productive natural forests are not managed in the same way as temperate forests or plantations, so modelling tools for carbon accounting developed for these forests (e.g. [28]-[30]) do not properly transfer to tropical realities. The current study provides a way to account for carbon in this context.

The break-even carbon price represents the minimum financial compensation a forest company would have to receive to change from the reference management to an improved management scenario [31]. This price is lower than what would actually generate a benefit from the carbon project. Considering that the timber contribution margin could vary between US$10 and US$40 m^−3^, the opportunity cost of carbon sequestration ranged between US$4.4 and US$25.9/ tCO_2_ depending on the management scenario implemented (Table 1 and considering that $\pi_{C}^{*}$ is proportional to *π*_*s*_). The range is broad, but it is consistent with the range of opportunity costs values commonly reported for forestry projects [13],[15],[16],[20],[21],[32]-[35]. Because there is no standard model to compute the opportunity cost, a direct comparison of opportunity cost values across studies seems irrelevant.

Our results are useful to identify some trends about opportunity costs for forest interventions in tropical countries. Many afforestation/reforestation projects have opportunity costs <US$8/ tCO_2_[11],[12]. For REDD+ projects in tropical forests, opportunity costs are also often <US$8/ tCO_2_, even if higher costs can occasionally be found when forest conservation is compared with highly profitable land-uses such as oil-palm cultivation [16],[21],[34].

Lengthening the felling cycle or raising cutting limits are not the only improved management practices that could be considered in a REDD+ porfolio. Adoption of RIL and silviculture practices could also be feasible REDD+ activities [19]. Contrary to changes of the management parameters that match the current logging practices, RIL implementation requires up-front capital investments in timber inventories, staff training, and sometimes new machinery, along with substantial modifications in working practices and monitoring [36]. Therefore, a REDD+ project based on RIL would require higher implementation costs than a REDD+ project based on changes of management parameters, and is perhaps less plausible in the short-term, at least in our study country. On the other hand, RIL-adoption may provide carbon revenues while maintaining timber revenues at a level close to the baseline. Considering that the same reasons why RIL has not been widely embraced may apply in the context of REDD+ [37], RIL was not the first improved management alternative that we considered.

Ours results question the representativeness of the Gabonese forest concession used for central African rain forests. The expected harvested timber volume in this operation (27 m^3^ ha^−1^) was greater than what is generally achieved in this region (<15 m^3^ ha^−1^, [9]), meaning that logging in our study was less selective than in the rest of the central African region. One effect of this intensive timber harvest is a high carbon break-even price. The initial stand biomass in the Haut-Abanga concession (360 t ha ^−1^) was within the range of values commonly found in the central African moist forests (i.e., 404 t ha ^−1^ with 348–488 t ha ^−1^ 95% confidence interval, according to [5]). In contrast, biomass was estimated to accumulate at high average rate of 2.8 t ha ^−1^ yr ^−1^ in the baseline scenario and at 3.1 t ha ^−1^ yr ^−1^ in the alternative scenarios. Because logging removes biomass through harvested timber and logging damage [38]-[40], for which we accounted in the model, the predicted biomass accumulation in the absence of logging is even greater (4.9 t ha ^−1^ yr ^−1^ on average across the first 40 yr). By comparison, Lewis et al. [5] found a mean biomass accumulation of 1.26 t ha ^−1^ yr ^−1^ (95% confidence interval: 0.44–1.88) in undisturbed forests of central Africa, whereas Gourlet-Fleury et al. [41] found a mean biomass accumulation of 4.82±1.22 t ha ^−1^ yr ^−1^ for logged plots in central Africa. Nevertheless, what matters for assessing the opportunity cost is not the biomass accumulation rate, but differences in accumulated biomass among management scenarios. An overestimated biomass accumulation rate does not necessarily result in an overvalued biomass benefit, but should this be the case, it would mean that the carbon break-even price was underestimated.

The high predicted biomass accumulation in the Haut-Abanga can be explained by an overestimated growth rate used for all species, and by the specific size-class distributions of some commercial species. The dbh growth rate used in this study (*a*=0.3 cm yr ^−1^) is the one defined in the Haut Abanga management plan. It is consistent with post-logging canopy gaps that boost growth but is greater than what is observed in undisturbed forests in central Africa (0.15–0.20 cm yr ^−1^ on average). Combined with a mortality rate of *m*=0.01 yr ^−1^, it corresponds to a mean tree dbh of 10+*a*/*m*=40 cm, thus much greater than the observed pre-harvest mean dbh of 27 cm. The dbh growth rate that would match this mean dbh value is 0.172 cm yr ^−1^. Nevertheless, because the break-even price of carbon is slightly sensitive to the growth rate, reducing *a* to 0.172 cm yr ^−1^ does not affect much the estimate of the break-even price (Additional file 2).

Regarding the dbh distributions, some dominant species in the Haut-Abanga concession have an unbalanced modal diameter distribution (e.g., pre-harvest dbh distribution of *Aucoumea klaineana* conforms to a normal distribution with mean 56 cm and standard deviation 26 cm, or *Scyphocephalium mannii* with mean 46 cm and std. dev. 18 cm). In the short-run, when the peak of the diameter distribution for these species increments, so does their total biomass because of the accumulation of large trees. In the longer-run, the peak vanishes and biomass decreases (e.g., the predicted total biomass for *A. klaineana* and *S. mannii* in the absence of logging decreases after 42 and 220 yr, respectively), which is not perceptible with a project length of 40 years. Nevertheless, given the low sensitivity of the break-even carbon price to both growth rates and dbh distributions, data on other more influential ecological parameters, in particular wood densities and species-specific allometric equations, should be improved.

Some simplifying assumptions that were made in our model could be relaxed to refine the estimated opportunity costs. For instance, the dependence of logging damage on logging intensity, and thus differences in logging damage between management scenarios, was not considered. We explored the consequences of relaxing this assumpion by replacing the constant damage rate of 10% by a function of the density of logged trees [42]. This dependence resulted in a reduction of the break-even price of carbon to US$8–9/tCO _2_ for a contribution margin of US$25 m ^−3^ (Additional file 2). This study was carbon-focused and did not consider non-carbon-related payments for ecosystem services [16]. The residual value of the forest at the end of the carbon project [43], or the feedback of the change of forest management on the timber market price were also disregarded. Nevertheless, these processes are likely to have marginal influences on carbon opportunity costs, without any a priori idea about whether this influence would be positive or negative. More importantly, we restricted the gain-loss analysis to the forest concessionaire, and did not integrate economic and environmental costs and benefits beyond timber production [44]. Integrating the whole chain of production and transformation, from logging to wood products export, would presumably provide other insights into carbon opportunity costs. However, as long as the articulation between the national scale for REDD+ project accounting and the local scale where carbon projects are implemented is not clarified, it is not clear what the cost-benefit analysis should encompass.

## Conclusions

At a global scale where multiple suppliers of emission reductions would compete in a REDD+ carbon market [34],[45],[46], tropical timber companies would change their management practices because of REDD+ opportunities only if very favourable conditions are met, namely if the timber contribution margin remains low enough and if alternative management practices and associated incentives are appropriately chosen. The current average price of carbon for improved forest management projects (US$10.4/ tCO_2_ in 2012 and US$10/tCO _2_ in 2011; [47],[48]) is unlikely to prompt forest concessionaires to forgo timber benefits. All in all, the approach we used to calculate the break-even price of carbon seems useful to inform on the available systems of incentives that could be part of the implementation of REDD+ mechanism as it pertains to different forest management scenarios. As such, the scenario analyses proposed represents only one of the elements of the still undefined REDD+ architecture (i.e. subnational and national programs) and unregulated markets for REDD+ credits. Other components related to governance (i.e. capacity of concessionaries to enforce their rights so as to exclude others from harvesting their timber and ability of governments to sanction those who illegally log and capture logging taxes), as well as other factors associated with delivery risks (e.g. mortality induced by drought, fire, and other disturbances) will determine the ultimate opportunity costs of policy scenarios based on contrasting forest management practices.

## Methods

### Study site and project scenario

The study was undertaken at the Haut-Abanga forest concession (288 627 ha) of the forest company Rougier-Gabon (between 10°30’–11°30’E in longitude and between 0°15’–0°50’N in latitude; [49]), at the western border of the Monts de Cristal mountain range. Altitudes vary between 250–1022 m. Climate is equatorial, with an annual rainfall between 1800–2000 mm depending on slope aspect, and mean annual temperatures between 24–26°C. The geological formation is an Archean basement with metamorphic and granitic rocks. Soils are mainly ferralitic sandy-clayey or clayey. The Haut-Abanga concession is covered with tropical moist forest, the dominant families being Burseraceae (18% of the basal area), Myristicaceae (15%), Caesalpiniaceae (15%) and Euphorbiaceae (9%) [49].

The management techniques considered in this study comply with the current legislation in Gabon that defines the technical norms for management of state-owned productive forests [50]-[52]. Accordingly, forest logging occurs periodically every *T* years, and between two successive logging operations, the forest is left for natural recovery. Because the forest concession is subsequently divided into quinquennial blocks, the length of the felling cycle *T* must be a multiple of 5 yr. Moreover, it must be greater than or equal to 20 yr. Logging of a commercial tree species *s* consists of removal of a fixed proportion of all trees with a diameter at breast height (dbh) greater than or equal to a cutting limit *d*_*s*_. This cutting limit must be greater than an administrative minimum cutting limit *A*_*s*_[53] (see Additional file 3 for species-specific values of *A*_*s*_). A management scenario is thus defined by the set of *S*+1 parameters (where *S* is the number of species), chosen by the forest concessionaire to comply with legal sustainability requirements: the length of the felling cycle *T* (the same for all species), and the dbh cutting limit *d*_*s*_ for each species *s*.

In this study, the baseline management scenario, denoted (*T*^ref^, $d_{s}^{\text{ref}}$), was defined by *T*^ref^=25 yr and $d_{s}^{\text{ref}}=A_{s}$. This baseline scenario corresponds to the current management plan of the Haut Abanga concession, which ensures that the stock recovery rate for each species is above its legal minimum. Two alternative scenarios (denoted *j*) were considered: (1) a project scenario (*T*, $d_{s}^{\text{ref}}$) in which *T*>*T*^ref^, with a longer felling cycle but the same cutting limits; and (2) a project scenario (*T*^ref^, *d*_*s*_) in which *d*_*s*_>*d* *s* ref, with higher cutting limits but the same length of the felling cycle (i.e., 25 yr). Because *T* must be a multiple of 5 yr and less than the project longevity, the possible values for *T* are limited: *T*=*T*^ref^+5, +10 or +15 yr. Because administrative cutting limits are multiple of 10 cm and because the forest inventory in Haut Abanga was correspondingly based on 10 cm-wide diameter classes, cutting limits were raised by +10, +20, +30 or +40 cm.

### Break-even price of carbon

The scenarios were compared to the baseline situation in which the felling cycle is 25 yr and the cutting limits are what is established by law (Additional file 3) by examining the carbon benefits in standing biomass and the associated timber losses (i.e. foregone timber harvest). Financial revenue from timber and carbon (i.e. credits for avoided carbon emissions) were computed over the duration *Ω* of the carbon project, which was equal to *Ω*=40 yr for this study. We defined ${\text{NPV}}_{T}^{\left(j\right)}$ and ${\text{NPV}}_{C}^{\left(j\right)}$ as the net present value of timber and carbon, respectively, according to project scenario *j*. Following the concept of additionality of the Kyoto Protocol [54], the carbon revenue for scenario *j* follows from the benefit of carbon storage for this scenario as compared to the baseline management scenario. Thus, by definition, ${\text{NPV}}_{C}^{\text{ref}}=0$. The break-even price of carbon sequestration (see definition in section “Opportunity cost” above) for project scenario *j* is:

1 $$\pi_{C}^{*}=\min\left\{\pi_{C}:{\text{NPV}}_{C}^{\left(j\right)}\geq {\text{NPV}}_{T}^{\text{ref}}-{\text{NPV}}_{T}^{\left(j\right)}\right\}$$

where *π*_*C*_ is the price of certificates of emission reductions (in US$ per tCO_2_) that defines the net present value of carbon. Future costs and benefits were discounted (discount rate: *δ*=12*%*) [55],[56], which is a minimum value for the private discount rate in the context of a forest industry in central Africa, where institutional stability is perceived as precarious and for an activity (logging) which is contested by environmental NGOs, making long term commercial prospects more uncertain when compared to other businesses.

#### Timber revenues

We here consider the standpoint of the concessionaire whose activity is to harvest and sell untransformed timber to a sawmill. Hence, even if the concessionaire is the owner of the sawmill, we do not consider revenues and costs associated with timber transformation. Let $X_{s}^{\left(j\right)}(t)$ be the harvested timber volume of species *s* at time *t* at the concession level under project scenario *j*. This timber volume brings a sale revenue of $\pi_{s}X_{s}^{\left(j\right)}(t)$, where *π*_*s*_ is the contribution margin (in US$ m ^−3^) for species *s*. The contribution margin here is the difference between the market price of untransformed timber times the proportion of timber that is not lost between the log yard and the mill entry, and all variable costs per unit of timber volume (including variable logging and transportation costs, and variable taxes). The net timber revenue is obtained after deduction of the fixed costs *Q*(*t*) associated with logging (including fixed taxes). The net present value of timber under project scenario *j* at the time horizon of the duration *Ω* of the carbon project thus is:

2 $${\text{NPV}}_{T}^{\left(j\right)}=\overset{\Omega }{\underset{t=1}{\sum }}\left\{\underset{s}{\sum }\pi_{s}X_{s}^{\left(j\right)}(t)-Q(t)\right\}{\left(1+\delta \right)}^{-t}$$

where the summation on *s* is over logged species only. Because fixed costs *Q*(*t*) theoretically do not depend on the management scenario *j*, they cancel out when computing the difference ${\text{NPV}}_{T}^{\text{ref}}-{\text{NPV}}_{T}^{\left(j\right)}$ and we do not have to estimate them to compute the break-even carbon price $\pi_{C}^{*}$.

The contribution margin *π*_*s*_ is difficult to assess because it depends on many interacting and fluctuating parameters (e.g., market prices, variable logging costs, transportation costs, variable taxes). Rather than fixing a contribution margin, we chose to consider it as a variable in the range of US$10–40 m ^−3^[57],[58] and to consider the break-even price $\pi_{C}^{*}$ as a function of *π*_*s*_ (the same for all commercial species). Having the same contribution margin *π* for all commercial species implies that $\pi_{C}^{*}$ is proportional to *π*.

#### Carbon revenues

There are many accounting methods for carbon, but two main approaches are distinguished [11],[29]. The first approach (the flow approach) is based on the flux of carbon entering the ecosystem and on the price of carbon within a period of time. The second approach (the stock approach) is based on a rent derived from stored carbon for a period of time. Following the VCS [25], we opted for the stock approach. The net carbon benefit (in tCO _2_ ha ^−1^) at time *t* for the *j* th management scenario with respect to the baseline scenario is:

3 $$\mathrm{\Delta C}(t)=C^{\left(j\right)}(t)-G^{\left(j\right)}(t)-\left\{C^{\text{ref}}(t)-G^{\text{ref}}(t)\right\}-L(t)$$

where *C*^(*j*)^(*t*) is the carbon stock (in tCO _2_ ha ^−1^) at the concession level, *G*^(*j*)^(*t*) is the greenhouse gas emissions (in tCO _2_ ha ^−1^) as a result of forest management activities, and *L*(*t*) is the greenhouse gas emissions (in tCO _2_ ha ^−1^) due to leakage. Leakage breaks down into leakage due to activity shifting, which is often assumed to be zero for improved forest management projects, and leakage due to market effects [25]. We here assumed that there was no market leakage (i.e, *L*(*t*)≃0). We also assumed that greenhouse gas emissions due to forest management activities were about the same for all management scenarios, so that *G*^(*j*)^(*t*) and *G*^ref^(*t*) cancelled out. Because *G*^(*j*)^(*t*) is likely to be <*G*^ref^(*t*), this simplifying assumption minimizes the risk of underestimating the break-even price of carbon, and is thus conservative.

Forest carbon is stored in living trees (both above- and below-ground), dead wood, litter, soil and wood products [25]. We assumed that the difference in below-ground biomass, necromass, litter biomass, or soil carbon among different management scenarios was <5% of the overall net carbon changes [59] and thus negligible with respect to the aboveground pool. Hence, these contributions to *C*^(*j*)^(*t*) canceled out when computing the difference *C*^(*j*)^(*t*)−*C*^ref^(*t*). Although there is potentially a large economic value associated with the carbon in long-lived wood products, we did not explicitly model these because when the timber is sold from the concession, the rights to capture any carbon value in wood products would presumably be also sold. Although a decision has been adopted at the COP 18 (Doha) to account for carbon storage in wood products, the issue of who, producer or product purchaser, could be credited for the carbon stored, remains unanswered. From the perspective of the land concessionaire, the economic value of carbon in wood products would be reflected by rising timber prices, which we explore in the sensitivity analysis. Therefore, the aboveground biomass is the only type of carbon storage that we considered.

Carbon credits were computed from the net carbon benefit using the VCS guidance for improved forest management projects with harvesting [24]. The long-term average of net carbon benefit was first computed over the duration of the project as: $A=\sum_{t=1}^{\Omega }\mathrm{\Delta C}(t)/\Omega$. This long-term average represents the number of carbon credits that correspond to permanent annual emission avoided if these were evenly distributed over the duration of the project. Because carbon benefits were actually not evenly distributed, the number of issuable carbon credits *U*(*t*) at time *t*≥1 was computed as the annual gain in net carbon benefit provided that this gain was positive and that the accumulated number of issuable carbon credits remained less than *A*:

4 $$U(t)=\max\left\{0;\min\left\{\mathrm{\Delta C}(t)-\mathrm{\Delta C}(t-1);A-\underset{s<t}{\sum }U(s)\right\}\right\}$$

This definition ensured that the total number of issuable carbon credits over the duration of the project equalled *A*: $\overset{\Omega }{\underset{t=1}{\sum }}U(t)=A$. To account for the non-permanence risk, the number of carbon credits issued at time *t* was finally determined as a fraction *ζ* of the issuable carbon credits, the remaining fraction 1−*ζ* being the proportion of carbon credits to be withheld as a buffer reserve. Following the risk analysis proposed by the VCS [60], we used *ζ*=78*%* (see Additional file 3). Thus, the net present value of carbon credits was:

5 $${\text{NPV}}_{C}^{\left(j\right)}=\pi_{C}\;\zeta \overset{\Omega }{\underset{t=1}{\sum }}U(t)\;{\left(1+\delta \right)}^{-t}$$

We did not consider any implementation cost (i.e. the cost of efforts needed to reduce deforestation and forest degradation) or transaction cost (i.e. the cost of establishing and operating the REDD+ project) when computing the net present value of carbon, which means that the break-even carbon price $\pi_{C}^{*}$ given by (1) represents the opportunity cost of carbon [31].

### Sensitivity analysis

To compute the break-even price of carbon, we calculated $X_{s}^{\left(j\right)}(t)$ and *C*^(*j*)^(*t*), the harvested timber volume of species *s*, and the carbon stock at time *t*, respectively. These values jointly derive from the state of the forest at that particular time, predicted using a model of forest dynamics based on a set of parameters that we hereafter refer to the ‘ecological’ parameters.

Because the same response pattern was obtained with sensitivity than with elasticity analyses, we here focus on the latter. The elasticity of the break-even price to a parameter *θ* is: $e_{\theta }=\partial \ln\overset{*}{\underset{C}{\pi }}/\partial \ln\theta$. The quantity *e*_*θ*_×*ξ* gives the proportional change of $\pi_{C}^{*}$ that would be brought by a small proportional perturbation of parameter *θ* in a proportion *ξ*. Elasticities can be added to assess the joint impact of several parameters. If ***θ***=(*θ*_1_,…,*θ*_*n*_) is a vector of *n* parameters, we define $e_{\theta }=\sum_{i=1}e_{\theta_{i}}$, so that *e*_***θ***_×*ξ* gives the proportional change of $\pi_{C}^{*}$ that would be obtained if all parameters *θ*_1_,…,*θ*_*n*_ were simultaneously changed by the same proportion *ξ*.

Economic parameters included in the sensitivity analysis are the species-specific contribution margins and the discount rate. In this case, the sensitivity analysis identifies the economic parameters that are to be considered at first to get the best control on the break-even price $\pi_{C}^{*}$. Ecological parameters are estimated from forest inventories or measured in the field, so they are not known with certainty. Estimation errors on these parameters bring an estimation error on the break-even price $\pi_{C}^{*}$. The sensitivity analysis in this case identifies the ecological parameters that should be estimated with the best precision to get the best precision on the estimated break-even price.

### Model of forest dynamics

The Technical National Guide (TNG) [52] does not recommend any model of forest dynamics, but the so-called stock recovery formula that it uses to assess sustainability implicitly relies on a simplified matrix projection model [61],[62]. This simplified model disregards recruitment and is thus inappropriate for long-term forecasts. For this reason, we used a matrix projection model that is simple enough to be consistent with the TNG, yet realistic enough to make long-term forecasts. This model is a Usher [26] matrix projection model with constant transition rates and a population growth rate equal to one. This latter assumption ensures that, in the absence of disturbance (such as logging), no population will indefinitely grow nor decline to extinction, and corresponds to the assumption that in mature undisturbed forests, species abundances remain approximately constant (at least over the mid-term range of our analyses).

The state of any tree species *s* at time *t* is defined by the per hectare numbers *N*_*is*_(*t*) of its individuals in *K* equal-width diameter classes. These per hectare numbers of trees are defined across operable areas of the forest concession only. Let **N**_*s*_(*t*)=[ *N*_1*s*_(*t*),…,*N*_*Ks*_(*t*)]^′^ be the *K*×1 column vector that compiles the per hectare number of trees in each diameter class for species *s*, and prime denotes the transpose. Its initial value **N**_*s*_(0) is provided by the management inventory. In the absence of disturbance, its temporal change is given by the recurrence formula:

6 $$N_{s}(t+1)=U_{s}\;N_{s}(t)$$

where **U**_*s*_ is a *K*×*K* Usher transition matrix with constant rates:

7 $$U_{s}=\left[\begin{array}{lllll} q_{s}\;+\;f_{s} & f_{s} & \cdots & f_{s} & f_{s}\\ p_{s} & q_{s} & 0\\ p_{s} & \ddots \\ \ddots & q_{s}\\ 0 & p_{s} & p_{s}\;+\;q_{s} \end{array}\right]$$

where *q*_*s*_, the stasis rate, represents the probability for a tree of species *s* to stay alive in the same diameter class between two successive time steps; *p*_*s*_, the upgrowth rate, represents the probability that a tree stays alive and moves up to the next diameter class between two successive time steps; and *f*_*s*_, the recruitment rate, represents the probability that a tree generates a newly recruited tree between two successive time steps. The population growth rate, *λ*_*s*_, corresponds to the dominant eigenvalue of the transition matrix **U**_*s*_[63]. For a matrix with constant rates like (4), it can be shown that: *λ*_*s*_=1+*f*_*s*_−*m*_*s*_, where *m*_*s*_=1−*q*_*s*_−*p*_*s*_ is the mortality rate for species *s*[62]. Assuming *λ*_*s*_=1 then is equivalent to assuming that the mortality and recruitment rates are equal.

Given *f*_*s*_=*m*_*s*_, matrix **U**_*s*_ can be reparameterized using only two transition rates, namely the mortality rate *m*_*s*_, and $p_{s}^{⋆}=p_{s}/(1-m_{s})$. Sometimes called the growth propensity, this latter rate represents the conditional probability that a tree moves up to the next diameter class knowing that it has stayed alive. It can be estimated as: $p_{s}^{⋆}=a_{s}\tau /\omega$, where *a*_*s*_ is the average diameter growth rate (in cm yr ^−1^) for species *s*, *τ* is the time interval (in yr) between two successive time steps, and *ω* is the width of the diameter classes (in cm). Hence, to compute the transition matrix **U**_*s*_ for species *s*, one only needs to know the mortality rate *m*_*s*_ and the diameter growth rate *a*_*s*_ for this species.

To complete the forest dynamics model, the temporal change of **N**_*s*_(*t*) when a disturbance occurs has to be specified. The only disturbance that we take into account is logging, assuming that the other disturbances impact the different management scenarios in a similar way. Logging is considered to be instantaneous with respect to forest dynamics. If logging occurs at time *t*, we distinguish the population state **N**_*s*_(*t*) before logging, and its state **N**_*s*_(*t*^+^) after logging. Logging harvests a proportion *ρ*_*s*_ of trees of species *s* with a dbh ≥*d*_*s*_. In addition to harvested trees, logging results in collateral damage and destruction of other trees. In the TNG, logging damage (i.e. trees destroyed by logging) is accounted by a proportion *ℓ*_*i*_ of trees that are removed in the *i* th diameter class when logging occurs. Hence, in matrix notation:

8 $$N_{s}(t^{+})=L\;H_{s}\;N_{s}(t)$$

where **L** is the *K*×*K* diagonal matrix whose *i* th element on the diagonal is 1−*ℓ*_*i*_, and **H**_*s*_ is the *K*×*K* diagonal matrix whose *i* th element on the diagonal is 1 if the upper bound of the *i* th diameter class is less than *d*_*s*_, and 1−*ρ*_*s*_ otherwise. Given that the first logging event occurs at time *c*, the complete description of forest dynamics is:

9 $$N_{s}(t+1,c)=\left\{\begin{array}{ll} U_{s}\;N_{s}(t,c) & \text{if}\;(t-c)\;\text{mod}\;(T/\tau )\neq 0\\ U_{s}\;L\;H_{s}\;N_{s}(t,c) & \text{if}\;(t-c)\;\text{mod}\;(T/\tau )=0 \end{array}\right.$$

where mod is the modulo operator, and *T*/*τ* gives the length of the felling cycle expressed as a number of time steps rather than in number of years. We added *c* as an argument to **N**_*s*_ to highlight the dependence on the logging schedule, given that **N**_*s*_(0,*c*)≡**N**_*s*_(0) is given by the initial forest inventory for all *c*.

The management inventory in the Haut-Abanga concession was conducted between 1998 and 2000, using a systematic sampling design based on 0.5-ha sampling plots, with a planned sampling rate of 1% for trees with dbh ≥20 cm and of 0.2% for trees with 10 cm < dbh ≤20 cm, and with an achieved rate of 1.2% for the former [49]. This forest inventory provided an estimate *N*_*is*_(0) at the concession level of the initial number of trees in *K*=16 diameter classes for *S*=313 morphospecies. Diameter classes are *ω*=10 cm wide, starting from 10 cm. Hence, the first class is 10–20 cm, the second class is 20–30 cm, till the sixteenth class that is ≥160 cm dbh. The mean diameter for the *i* th diameter class was computed as *D*_*i*_=(10*i*+5) cm. The pre-harvest dbh distribution of the forest had a typical reverse-J shape that conformed to an exponential distribution with parameter 0.058 cm ^−1^. The 313 morphospecies were assigned by Rougier-Gabon to seven groups: six commercial groups by decreasing order of commercial importance, and one group of protected species (Additional file 3). In this study, we considered that only groups 1 and 2 were logged. Group 1 contains a single species (*Aucoumea klaineana* Pierre, that represents 80% of the wood production in Gabon), and group 2 contains 38 morphospecies. The logging intensity for all management scenarios was *ρ*_*s*_=90*%* for all logged species. The time step of the matrix model was *τ*=1 year. To comply with the dynamics parameters used in the management plan of the Haut-Abanga, we used for all species a dbh growth rate of *a*_*s*_=3 mm yr ^−1^ and a mortality rate of *m*_*s*_=1*%*, which means that the stasis rate was 96.03% while the upgrowth transition rate was 2.97%. The management plan at the Haut-Abanga was based on a constant logging damage rate of 10% [64]. Consistently, and considering that the rate of trees destroyed by logging decreases with tree size [42], we used a logging damage of 10% for the first three classes, then null: *ℓ*_*i*_=0.1 for *i*≤3 and *ℓ*_*i*_=0 for *i*≥4.

#### Timber volume dynamics

Timber volume dynamics were obtained by converting tree diameter into volume, using volume equations taken from the management plan of the forest concession when available, or, by default, from the TNG. These are species-specific equations that predict the volume of a tree from its dbh, and are given in Additional file 3. Given that the first logging event occurs at time *c*, the population-level estimate of harvested timber volume for species *s* at time *t* is:

10 $$W_{s}(t,c)=\left\{\begin{array}{ll} 0 & \text{if}\;(t-c)\;\text{mod}\;(T/\tau )\neq 0\\ V_{s}^{\prime }(I-H_{s})\;N_{s}(t,c) & \text{if}\;(t-c)\;\text{mod}\;(T/\tau )=0 \end{array}\right.$$

where **V**_*s*_ is the *K*×1 column vector [ *V*_*s*_(*D*_1_),…,*V*_*s*_(*D*_*K*_)]^′^, *V*_*s*_ is the volume equation for species *s*, *D*_*i*_ is the mean diameter for the *i* th diameter class, and **I** is the *K*×*K* identity matrix. The total harvested wood volume at time *t* is obtained by summing *W*_*s*_(*t*,*c*) over all logged species (disregarding unlogged species).

The population-level estimates of timber volume were summed across annual cutting units to estimate timber volume at the concession level. Because the forest concession is divided into quinquennial blocks that are in turn divided into annual cutting units, we consider that at every time step a proportion *τ*/*T* of the concession is logged. Hence, at the spatial scale of a cutting unit, there is a cyclic succession of logging events every *T* years, whereas at the concession level, logging occurs continuously. The harvested timber volume for species *s* at time *t* at the concession level under management scenario *j* thus is:

11 $$X_{s}^{\left(j\right)}(t)=\frac{\tau }{T}\overset{T/\tau }{\underset{c=1}{\sum }}W_{s}^{\left(j\right)}(t,c)$$

where $W_{s}^{\left(j\right)}$ is computed using (6) under the *j* th management scenario, and *c* indexes cutting units within the forest concession, considering that the first unit is initially logged, that the second unit is logged in year *τ*, and so on till the (*T*/*τ*)th unit that is logged in year *T*−*τ*.

#### Carbon dynamics

Carbon dynamics were obtained by converting tree diameter into aboveground carbon, using an allometrical biomass equation. As carbon issues are currently not addressed by the TNG, no biomass equations were recommended by the TNG. Hence, we used Chave et al. [65] commonly used equation for tropical moist forests. The aboveground biomass (in kg) for species *s* at time *t* thus is:

12 $$M_{s}(t,c)=w_{s}\;B^{\prime }\;N_{s}(t,c)$$

where *w*_*s*_ is the wood density (in g cm ^−3^) for species *s*, and **B** is the *K*×1 column vector [ *B*(*D*_1_),…,*B*(*D*_*K*_)]^′^ defined by *B*(*D*_*i*_)= exp{−1.499+2.1481 ln(*D*_*i*_)+0.207[ ln(*D*_*i*_)]^2^−0.0281[ ln(*D*_*i*_)]^3^}, where *D*_*i*_ is expressed in cm. The specific wood densities were taken from the Zanne et al. [66] database. When no match at the species level was found, an average value at the genus level was taken. When no match at the genus level was found in the database, the default value of 0.60 g cm^−3^ recommended by Henry *et al.* ([67], p.1383) for tropical African woods was taken. Species-specific wood densities are listed in Additional file 3.

The population-level dry aboveground biomass at time *t* is obtained by summing *M*_*s*_(*t*,*c*) over all species (including unlogged species). As for timber volumes, this value is then summed across annual cutting units to get the dry aboveground biomass at the concession level. Dry biomass is converted into CO _2_ equivalents using fixed conversion rates. Adding all the carbon pools, the carbon stock (in tons of CO _2_) at time *t* at the concession level under management scenario *j* is:

13 $$C^{\left(j\right)}(t)=\gamma^{-1}\alpha \overset{S}{\underset{s=1}{\sum }}\frac{\tau }{T}\overset{T/\tau }{\underset{c=1}{\sum }}M_{s}^{\left(j\right)}(t,c)+C_{\text{prod}}^{\left(j\right)}(t)+C_{\text{other}}^{\left(j\right)}(t)$$

where *γ*=12/44 ton of C per ton of CO_2_ is the mass proportion of carbon in CO_2_, *α*=0.47 ton of C per ton of biomass is the conversion rate from biomass to carbon (Table 4.3 in [68]), the summation on *s* is over all species (including unlogged species), $M_{s}^{\left(j\right)}$ is computed using (8) under the *j* th management scenario, *C*_prod_ is the carbon stock in long-lived wood products, and *C*_other_ is the carbon stock in the other pools (below-ground biomass and necromass).

## Additional files

## Electronic supplementary material

Additional file 1: **Detailed results of the elasticity analysis.** The additional file contains detailed results of the elasticity analysis of the break-even price of carbon to ecological parameters. (PDF 178 KB)

Additional file 2: **Break-even price for different management parameters.** The additional file contains estimations of the break-even price of carbon when different values of the management parameters are used. (PDF 126 KB)

Additional file 3: **Model parameters.** The additional file contains the list of mathematical symbols, the risk analysis to determine the number of buffer credits, and the species specific parameters and volume equations. (PDF 167 KB)

Below are the links to the authors’ original submitted files for images.

Authors’ original file for figure 1

Authors’ original file for figure 2

Authors’ original file for figure 3
