# Supplementary material for: Opportunity costs of carbon sequestration in a forest concession in central Africa
Source: Carbon Balance Manag. 2014 Jul 3;9:4. doi: 10.1186/s13021-014-0004-3 (PMC4637000; doi:10.1186/s13021-014-0004-3)
Supplement: Supplementary file 1 — Additional file 1: Detailed results of the elasticity analysis. The additional file contains detailed results of the elasticity analysis of the break-even price of carbon to ecological parameters. (PDF 178 KB) [file 13021_2014_4_MOESM1_ESM.pdf]

Additional file 1

Opportunity cost of carbon sequestration with respect to timber production in a forest concession in central Africa: Detailed results of the elasticity analysis

Michel Ndjondo, Sylvie Gourlet-Fleury, Raphaël J. Manlay, Nestor Laurier Engone Obiang, Alfred Ngomanda, Claudia Romero, Florian Claeys, Nicolas Picard*
*Corresponding author: nicolas.picard@cirad.fr

Elasticities of the break-even price of carbon to ecological parameters

The sensitivity of the break-even price π_C^* of carbon to a parameter θ is:

$$\sigma_\theta = \frac{\partial \pi_C^*}{\partial \theta}$$

whereas the elasticity of the break-even price to this parameter is:

$$e_\theta = \frac{\partial \ln \pi_C^*}{\partial \ln \theta} = \left(\frac{\partial \pi_C^*}{\partial \theta}\right)\left(\frac{\theta}{\pi_C^*}\right)$$

$\sigma_\theta \times \Delta$ gives the amount by which π_C^* changes if parameter θ is changed by a small additive perturbation Δ. $e_\theta \times \xi$ gives the proportional change of π_C^* that is brought by a small proportional perturbation of parameter θ in a proportion ξ. Sensitivities and elasticities can be added to assess the joint impact of several parameters. If $\theta = (\theta_1, \ldots, \theta_n)$ is a vector of n parameters, we define $\sigma_\theta = \sum_{i=1}^n \sigma_{\theta_i}$ and $e_\theta = \sum_{i=1} e_{\theta_i}$. Then, $\sigma_\theta \times \Delta$ gives the amount by which π_C^* changes if all parameters $\theta_1, \ldots, \theta_n$ are simultaneously changed by the same additive perturbation Δ, whereas $e_\theta \times \xi$ gives the proportional change of π_C^* that is obtained if all parameters $\theta_1, \ldots, \theta_n$ are simultaneously changed in the same proportion ξ.

The break-even price is computed for the project scenario with a longer rotation $T^{\text{ref}} + 10$ yr. Expectedly, the elasticities to the specific densities w_s and to the class-dependent biomasses $B(D_i)$ are all negative (Fig.S2-1B, C) since an increase in these parameters brings an increase in the net carbon benefit and consequently a decrease in the break-even price of carbon, whereas the elasticities to $V_s(D_i)$ are all positive (Fig.S2-1E, F) since an increase in these parameters brings an increase in PVT. Most of the elasticities to mortality rates m_s are positive (Fig.S2-1I). The elasticities to the growth rates a_s (Fig.S2-1D) and to the initial number of trees $N_{is}(0)$ (Fig.S2-1G, H) are positive or negative depending on the species and the diameter class. These changing signs reflect the influence of the shape of the diameter distribution. For species like *Aucoumea klaineana* that have a hump-shaped diameter distribution, with many large trees and a deficit of juveniles, increasing the growth rate intensifies the "primary forest premium" effect. For those parameters that are species-specific (Fig.S2-1, right column), the S species

impact differently the break-even price, with *A. klaineana* always standing as the species with the greatest impact on π_C^*. For those parameters that vary with the diameter class (Fig.S2-1C, E, G), the K classes also impact differently the break-even price, with one peak (in absolute value) around class 2–3 and/or another peak around class 7–8 (that is close to the cutting limit).

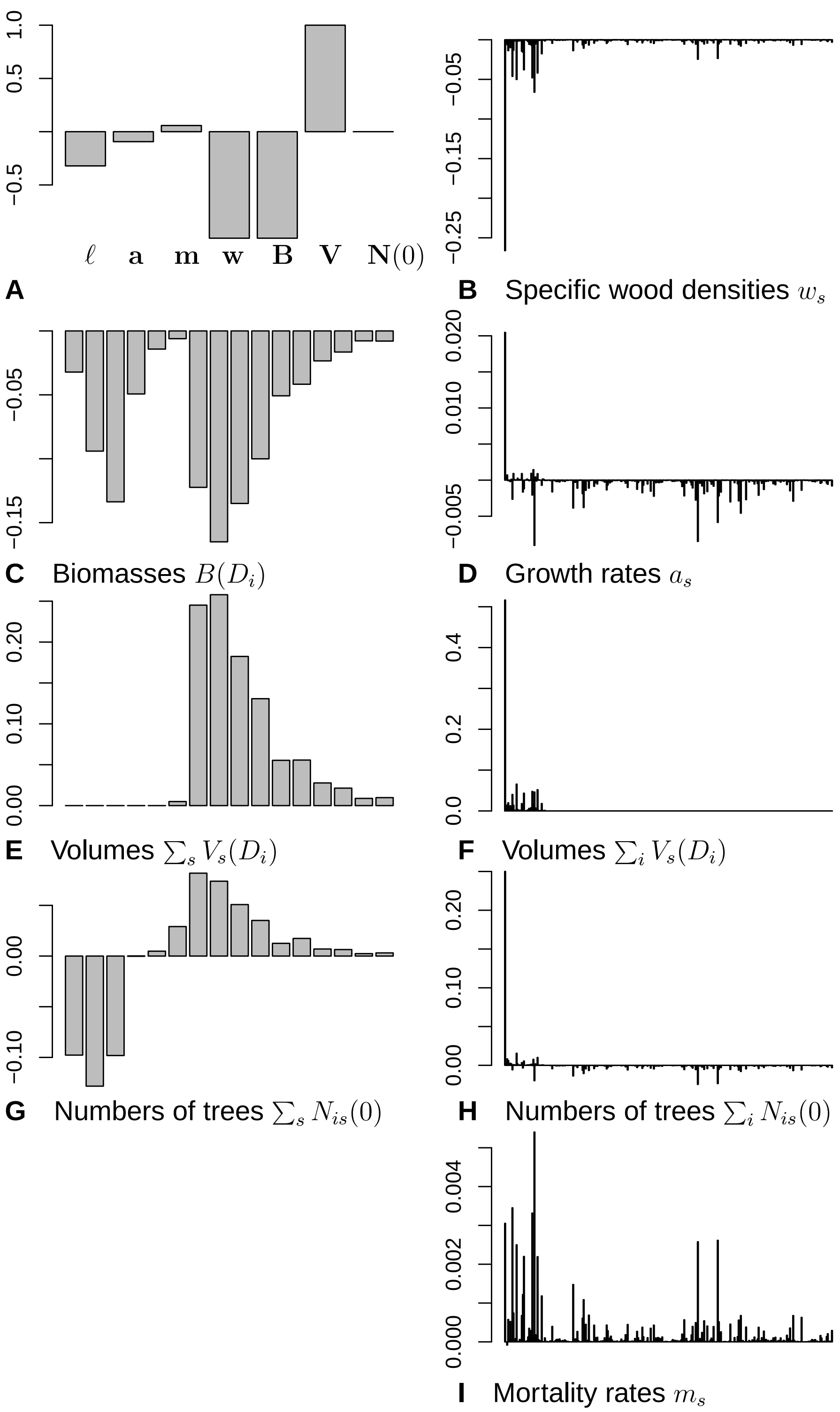

Fig. S2-1: Elasticities of the break-even price π_C^* of carbon to ecological parameters. A. Elasticities to vectors of parameters, where $\ell =$ logging damages, $\mathbf{a} = (a_s)_{s=1\ldots S} =$ growth rates, $\mathbf{m} = (m_s)_{s=1\ldots S} =$ mortality rates, $\mathbf{w} = (w_s)_{s=1\ldots S} =$ wood densities, $\mathbf{B} = [B(D_i)]_{i=1\ldots K} =$ class-dependent biomasses, $\mathbf{V} = [V_s(D_i)]_{s=1\ldots S, i=1\ldots K} =$ species- and class-dependent volumes, and $\mathbf{N}(0) = [N_{is}(0)]_{i=1\ldots K, s=1\ldots S} =$ initial numbers of trees. B–I clarify the elasticities of π_C^* to each element of these vectors, with species-dependent parameters on the right, and class-dependent parameters on the left. For species-dependent parameters, there are $S = 313$ bars and *Aucoumea klaineana* corresponds to the first bar. For class-dependent parameters, there are $K = 16$ bars corresponding to the K diameter classes.
