# Supplementary material for: Opportunity costs of carbon sequestration in a forest concession in central Africa
Source: Carbon Balance Manag. 2014 Jul 3;9:4. doi: 10.1186/s13021-014-0004-3 (PMC4637000; doi:10.1186/s13021-014-0004-3)
Supplement: Supplementary file 2 — Additional file 2: Break-even price for different management parameters. The additional file contains estimations of the break-even price of carbon when different values of the management parameters are used. (PDF 126 KB) [file 13021_2014_4_MOESM2_ESM.pdf]

Additional file 2

Opportunity cost of carbon sequestration with respect to timber production in a forest concession in central Africa: Break-even price of carbon for different management parameter values

Michel Ndjondo, Sylvie Gourlet-Fleury, Raphaël J. Manlay, Nestor Laurier Engone Obiang, Alfred Ngomanda, Claudia Romero, Florian Claeys, Nicolas Picard*
*Corresponding author: nicolas.picard@cirad.fr

In this additional file, we redo the computation of the break-even price of carbon with different values of the management parameters, to assess the robustness of the estimate of the break-even price.

1 Break-even price of carbon when damage rate varies across management scenarios

By default, we used a constant logging damage rate of 10%, irrespective of management scenario. To relax this simplifying assumption, we also computed the break-even price of carbon when logging damage depends on the density of logged trees. We used the relationship given by Picard et al. (2012) between logging damage ℓ (dimensionless) and the density of logged trees h (in stems ha^{-1}):

$$\ell = 1 - \frac{1}{(1+0.09135h)^{0.7046}}$$

where h is computed from $\mathbf{N}_s$ using: $h = \sum_s \mathbf{1}'(\mathbf{I}-\mathbf{H}_s)\mathbf{N}_s$, where the sum on s is over commercial species only, and $\mathbf{1}$ is the vector of length K full of ones (see section "Model of forest dynamics" of the manuscript). The resulting break-even price of carbon for a contribution margin of US\$25/m^3 is given in Table S3-1.

2 Break-even price of carbon for a discount rate of 8%

By default, we used of discount rate of 12%. When the discount rate is lowered to 8%, the break-even price of carbon is slightly modified (Table S3-2), which is consistent with the result of the sensitivity analysis that showed that the break-even price of carbon was slightly sensitive to the discount rate.

Tab. S3-1: Opportunity cost of carbon sequestration for different alternative management scenarios in the Haut-Abanga concession, when logging damage is a function of the density of logged trees. The break-even price of carbon sequestration π_C^* is computed for a contribution margin of US\$25/m^3 for all commercial species. $\sum$Credits is the sum of carbon credits. ΔVolume is the total reduction in harvested wood volume. ΔPVT_T is the loss in the net present value of timber.

Scenario	ΔVolume ($m^3 ha^{-1}$)	$\sum$Credits ($tCO_2 ha^{-1}$)	ΔPVT_T (US\$/ha)	π_C^* (US\$/tCO_2)
Lengthened rotation (+5 yr)	1.8	8.5	34.2	8.9
Lengthened rotation (+10 yr)	3.3	16.0	60.5	8.9
Lengthened rotation (+15 yr)	4.7	22.7	81.2	8.8
Raised cutting limits (+10 cm)	8.9	22.6	65.5	7.9
Raised cutting limits (+20 cm)	17.5	41.9	124.2	8.1
Raised cutting limits (+30 cm)	24.5	55.3	166.2	8.4
Raised cutting limits (+40 cm)	29.5	63.5	193.6	8.6

Tab. S3-2: Opportunity cost of carbon sequestration for different alternative management scenarios in the Haut-Abanga concession, for a discount rate of 8%. The break-even price of carbon sequestration π_C^* is computed for a contribution margin of US\$25/m^3 for all commercial species. $\sum$Credits is the sum of carbon credits. ΔVolume is the total reduction in harvested wood volume. ΔPVT_T is the loss in the net present value of timber.

Scenario	ΔVolume ($m^3 ha^{-1}$)	$\sum$Credits ($tCO_2 ha^{-1}$)	ΔPVT_T (US\$/ha)	π_C^* (US\$/tCO_2)
Lengthened rotation (+5 yr)	1.8	7.1	41.6	10.5
Lengthened rotation (+10 yr)	3.3	13.0	75.3	10.7
Lengthened rotation (+15 yr)	4.7	18.2	102.9	10.9
Raised cutting limits (+10 cm)	8.9	10.3	89.4	16.7
Raised cutting limits (+20 cm)	17.5	19.5	170.4	16.9
Raised cutting limits (+30 cm)	24.5	26.3	229.9	17.1
Raised cutting limits (+40 cm)	29.5	30.7	269.5	17.3

3 Break-even price of carbon for a growth rate of 0.172 cm yr^{-1}

By default, we used a dbh growth rate of $a = 0.3$ cm yr^{-1}, which corresponds to a dbh distribution at steady state (without logging) that is exponential with parameter $m/a = 0.033$ cm^{-1}, where $m = 1\%$ yr^{-1} is the default value of the mortality rate. The pre-harvest dbh distribution in the Haut Abanga concession was exponential with parameter 0.058 cm^{-1}, which corresponds to a dbh growth rate of $a = 0.172$ cm yr^{-1}. When the dbh growth rate is lowered from 0.3 to 0.172 cm yr^{-1}, the carbon stock has an overall trend that is decreasing (Figure S3-1). However, the break-even price of carbon is slightly modified (Table S3-3), which is consistent with the result of the sensitivity analysis that showed that the break-even price of carbon was slightly sensitive to the dbh growth rate.

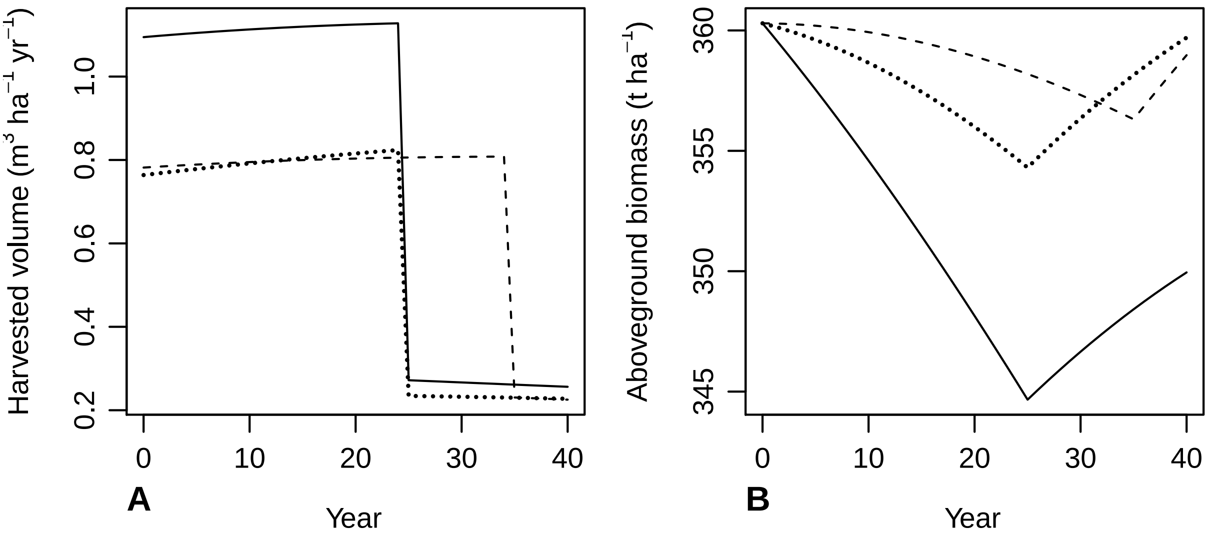

Fig. S3-1: Temporal forecasts in the Haut-Abanga forest concession, Gabon, when using a dbh growth rate of 0.172 cm yr^{-1}. Harvested wood volume (panel A) and aboveground biomass (panel B) are forecast according to three management scenarios: solid line is the reference scenario (T^{ref}, d_s^{ref}); dashed line is an alternative scenario with a longer felling cycle $T = T^{\mathrm{ref}} + 10$ yr; and dotted line is an alternative scenario with higher cutting limit diameters $d_s = d_s^{\mathrm{ref}} + 10$ cm.

Tab. S3-3: Opportunity cost of carbon sequestration for different alternative management scenarios in the Haut-Abanga concession, for a dbh growth rate of 0.172 cm yr^{-1}. The break-even price of carbon sequestration π_C^* is computed for a contribution margin of US\$25/m^3 for all commercial species. $\sum$Credits is the sum of carbon credits. ΔVolume is the total reduction in harvested wood volume. ΔPVT$_T$ is the loss in the net present value of timber.

Scenario	ΔVolume (m^3ha^{-1})	$\sum$Credits (tCO_2ha^{-1})	ΔPVT$_T$ (US\$/ha)	π_C^* (US\$/tCO_2)
Lengthened rotation (+5 yr)	1.5	5.9	32.1	11.6
Lengthened rotation (+10 yr)	2.7	10.8	56.7	11.6
Lengthened rotation (+15 yr)	3.9	15.1	75.9	11.6
Raised cutting limits (+10 cm)	8.5	9.2	63.3	16.3
Raised cutting limits (+20 cm)	16.3	17.3	119.2	16.4
Raised cutting limits (+30 cm)	22.2	23.1	157.6	16.4
Raised cutting limits (+40 cm)	26.1	26.7	182.2	16.5

References

Picard, N., S. Gourlet-Fleury, and E. Forni, 2012. Estimating damage from selective logging and implications for tropical forest management. *Canadian Journal of Forest Research* **42**:605–613.
