# Supplementary material for: Opportunity costs of carbon sequestration in a forest concession in central Africa
Source: Carbon Balance Manag. 2014 Jul 3;9:4. doi: 10.1186/s13021-014-0004-3 (PMC4637000; doi:10.1186/s13021-014-0004-3)
Supplement: Supplementary file 3 — Additional file 3: Model parameters. The additional file contains the list of mathematical symbols, the risk analysis to determine the number of buffer credits, and the species specific parameters and volume equations. (PDF 167 KB) [file 13021_2014_4_MOESM3_ESM.pdf]

Additional file 3

Opportunity cost of carbon sequestration with respect to timber production in a forest concession in central Africa: Model parameters

Michel Ndjondo, Sylvie Gourlet-Fleury, Raphaël J. Manlay, Nestor Laurier Engone Obiang, Alfred Ngomanda, Claudia Romero, Florian Claeys, Nicolas Picard*
*Corresponding author: nicolas.picard@cirad.fr

1 List of mathematical symbols

1.1 Latin symbols

a_s: the average diameter growth rate (in $\mathrm{cm\,yr^{-1}}$) for species s
A: long-term average of net carbon benefit
A_s: administrative minimum cutting limit for species s
$\mathbf{B}$: the biomass vector = $K \times 1$ column vector $[B(D_1), \ldots, B(D_K)]'$
$B(D)$: aboveground dry biomass of a tree with dbh D and whose wood density equals 1 $\mathrm{g\,cm^{-3}}$
c: time index for a logging event; correspondingly, c also indexes annual cutting units within the forest concession (identifying the 1st annual cutting unit as the one that is cut at year 1, the 2nd unit as the one that is cut at year 2, etc.)
$C(t)$: carbon stock at time t at the concession level
C_{prod}: carbon stock in long-lived wood products
C_{other}: carbon stock in other compartments than standing aboveground biomass and long-lived wood products
D_i: the mean diameter for the ith diameter class
d_s: cutting limit dbh for species s
e_θ: the elasticity of the break-even price π_C to parameter θ
f_s: the recruitment rate for species s = the probability for a tree of species s to generate a newly recruited tree between two successive time steps
$G(t)$: greenhouse gas emission at time t as a result of forest management activities
h: density of logged trees
$\mathbf{H}_s$: the harvest matrix = $K \times K$ diagonal matrix whose ith element on the diagonal is 1 if the upper bound of the ith diameter class is less than d_s, and $1-\rho_s$ otherwise
i: index of a diameter class ($1 \le i \le K$)
$\mathbf{I}$: the $K \times K$ identity matrix
j: index of a management scenario
K: number of diameter classes
ℓ_i: logging damage rate in diameter class i = proportion of trees that are removed due to damage in the ith diameter class when logging occurs
$L(t)$: greenhouse gas emission at time t due to leakage

L: the logging damage matrix = $K \times K$ diagonal matrix whose ith element on the diagonal is ℓ_i

m_s: mortality rate for species s = the probability for a tree of species s to die between two successive time steps

$M_s(t,c)$: cumulative biomass for species s at time t, given that logging occurred at time c

$\mathbf{N}_s(0)$: $K \times 1$ column vector $[N_{1s}(0), \ldots, N_{Ks}(0)]'$

n: index of a parameter of the model

$N_{is}(0)$: the initial number of trees of species s in the ith diameter class, as provided by the management inventory

$\mathbf{N}_s(t,c)$: $K \times 1$ column vector $[N_{1s}(t,c), \ldots, N_{Ks}(t,c)]'$

$N_{is}(t,c)$: the number of individuals of species s in diameter class i at time t, given that logging occurred at time c

$\mathrm{NPV}_{\mathrm{C}}^{(j)}$: net present value of carbon under project scenario j

$\mathrm{NPV}_{\mathrm{T}}^{(j)}$: net present value of timber under project scenario j

$p_s^\star$: growth propensity $= p_s/(1-m_s)$ = the conditional probability for a tree of species s to move up to the next diameter class between two successive time steps knowing that it has stayed alive

p_s: the upgrowth rate = the probability for a tree of species s to stay alive and move up to the next diameter class between two successive time steps

q_s: the stasis rate = the probability for a tree of species s to stay alive in the same diameter class between two successive time steps

Q: fixed costs of logging (including fixed taxes)

r: proportion of timber that is lost between the log yard and the mill entry

s: index of a species ($1 \leq s \leq S$)

S: number of species

t: index of time

T: the rotation = the length of the felling cycle (must be a multiple of 5 years)

(T, d_s^{ref}): management scenario where $T > T^{\mathrm{ref}}$, with a longer felling cycle but the same cutting limits

$(T^{\mathrm{ref}}, d_s^{\mathrm{ref}})$: reference management scenario

(T^{ref}, d_s): management scenario where $d_s > d_s^{\mathrm{ref}}$, with higher cutting limits but the same rotation

$U(t)$: number of issuable carbon credits at time t

$\mathbf{U}_s$: $K \times K$ Usher transition matrix for species s

$\mathbf{V}$: $(S \times K)$-vector $(\mathbf{V}_1, \ldots, \mathbf{V}_S)$

$\mathbf{V}_s$: $K \times 1$ column vector $[V_s(D_1), \ldots, V_s(D_K)]'$

$V_s(D)$: volume of a tree with species s and dbh D

$\mathbf{w}$: S-vector $(w_1, \ldots, w_S)$

w_s: wood density (in $\mathrm{g\,cm^{-3}}$)

$W_s(t,c)$: harvested timber volume for species s at time t, given that logging occurred at time c

$X_s(t)$: harvested timber volume for species s at time t at the concession level

1.2 Greek symbols

α: conversion rate from dry biomass to carbon = 0.47 ton of C per ton of dry biomass

β_s: variable costs (including variable logging costs, transportation costs, and variable taxes) for species s

γ: mass proportion of carbon in CO_2 = 0.273 ton of C per ton of CO_2

δ: discount rate

Δ: a small additive change of a parameter value
$\Delta C(t)$: net carbon benefit (in tCO_2 ha^{-1}) at time t with respect to the reference scenario
ζ: fraction of issuable carbon credits that can be issued, the remaining fraction being withheld as a buffer reserve for the non-permanence risk
$\boldsymbol{\theta}$: a vector of parameters of the model
θ: a parameter of the model
λ_s: population growth rate for species s
ξ: a small proportional change of a parameter value
π: S-vector $(\pi_1, \ldots, \pi_S)$ of the specific contribution margins
π_C: the price of certificates of emission reductions (in US$ per tCO_2)
π_C^*: the break-even price of carbon credits (in US$ per tCO_2)
π_s: contribution margin for species s
ϖ_s: market price of untransformed timber for species s
ρ_s: logging intensity = proportion of all trees with a dbh greater than or equal to d_s that are harvested by logging
σ_θ: the sensitivity of the break-even price π_C to parameter θ
τ: time interval (in yr) between two successive time steps
ω: the width of the diameter classes (in cm)
Ω: duration of the carbon project (in yr; must be a multiple of 5 years)

1.3 Non-alphabetic symbols

$\mathbf{1}$: vector of length K full of ones

2 Risk analysis for an improved forest management REDD+ project in Haut Abanga, Gabon

The risk analysis aims at determining the non-permanence risk rating, which shall be used to determine the number of buffer credits that the project shall deposit into a pooled buffer account. The risk analysis was conducted following the VCS non-permanence risk tool version 3.2 (Verified Carbon Standard, 2012) and yielded an overall risk rating of 22 (Table S1-1). In comparison, TEREA (2013) for a virtual improved forest management REDD+ project in the Haut Nyong, Cameroon, obtained an overall risk rating of 24. For the Maï Ndombé REDD+ project in the Democratic Republic of Congo (project #934 in the VCS project database), an overall risk rating of 25 was used. For the Pikounda REDD+ project in Congo (project #1052 in the VCS project database), an overall risk rating of 21 was used.

Tab. S1-1: Risk analysis at Haut Abanga, Gabon.

	Risk	Value	Score	Explanation
A. Internal risks				
A1. Project management risks				
a)	Species planted (where applicable) associated with more than 25% of the stocks on which GHG credits have previously been issued are not native or proven to be adapted to the same or similar agro-ecological zone(s) in which the project is located.	2	0	The Haut Abanga forest is a natural forest with no plantations that contribute to the project.

	Risk	Value	Score	Explanation
b)	Ongoing enforcement to prevent encroachment by outside actors is required to protect more than 50% of stocks on which GHG credits have previously been issued.	2	0	The Haut Abanga forest is an isolated area with no outside actors nearby.
c)	Management team does not include individuals with significant experience in all skills necessary to successfully undertake all project activities (ie, any area of required experience is not covered by at least one individual with at least 5 years experience in the area).	2	0	Forest concessionaires are already trained on sustainable forest management. Lengthening the felling cycle or raising diameter cuting limits does not require new skills.
d)	Management team does not maintain a presence in the country or is located more than a day of travel from the project site, considering all parcels or polygons in the project area.	2	0	The forest concessionaire maintains a permanent presence in the project area and in Libreville (less than a day of travel fromt the site).
e)	Mitigation: Management team includes individuals with significant experience in AFOLU project design and implementation, carbon accounting and reporting (eg, individuals who have successfully managed projects through validation, verification and issuance of GHG credits) under the VCS Program or other approved GHG programs.	−2	0	The forest concessionaire is not well trained on REDD+ projects.
f)	Mitigation: Adaptive management plan in place.	−2	0	Current forest management plans are not adaptive (in the sense given by VCS).
	Total for project management		0	
	A2. Financial viability risks			
a-d)	Project cash flow breakeven point greater than 4 and up to 7 years from the current risk assessment	0–3	1	The project would include support from donors interested in the development of alternative forest management practices.
e-h)	Project has secured 40% to less than 80% of funding needed to cover the total cash out required before the project reaches breakeven	0–3	1	Forest concessionaires are cautious regarding REDD+ projects and would not run into them without ensuring that enough funding is available.
i)	Mitigation: Project has available as callable financial resources at least 50% of total cash out before project reaches breakeven	−2	0	
	Total for financial viability		2	
	A3. Opportunity costs risks			
a-f)	NPV from the most profitable alternative land use activity is expected to be at least 100% more than that associated with project activities; or where baseline activities are subsistence-driven, net positive community impacts are not demonstrated	0–8	8	The NPV were computed on the basis of a contribution margin of $\pi_s = 25$ US$/m^3 for timber and of a carbon price of 12,7 US$/tCO$_2$.
g)	Mitigation: Project proponent is a non-profit organization	−2	0	The project proponent is from the private sector.

	Risk	Value	Score	Explanation
h-i)	Mitigation: Project is protected by legally binding commitment (see Section 2.2.4) to continue management practices that protect the credited carbon stocks over the length of the project crediting period. Or: Project is protected by legally binding commitment (see Section 2.2.4) to continue management practices that protect the credited carbon stocks over at least 100 years	−2	−2	The FSC certification of the Haut Abanga concession could possibly ensure that the improved management practices are continued over the length of the project period.
	Total for opportunity costs		6	
	A4. Project longevity risks			
b)	With legal agreement or requirement to continue the management practice	$30-L/2$	10	Considering that the FSC certification is a legal agreement and that the project longevity (L, in years) is 40 years.
	Total for project longevity		10	
	Total for internal risks		18	($\leq$ 35 for eligibility)
B. External risks				
	B1. Land tenure and resource access/impacts risks			
a-b)	Ownership and resource access/use rights are held by different entity(s)	0–2	2	Land is government owned and the project proponent holds a lease or concession
c)	In more than 5% of the project area, there exist disputes over land tenure or ownership	10	0	There is no dispute over land tenure or ownership in Haut Abanga
d)	There exist disputes over access/use rights (or overlapping rights)	5	0	Mining can generate dispute over access/use rights in forest concessions in Gabon, but there is no mining permit in Haut Abanga.
e)	WRC projects unable to demonstrate that potential upstream and sea impacts that could undermine issued credits in the next 10 years are irrelevant or expected to be insignificant, or that there is a plan in place for effectively mitigating such impacts.	2	0	Not applicable.
f)	Mitigation: Project area is protected by legally binding commitment (eg, a conservation easement or protected area) to continue management practices that protect carbon stocks over the length of the project crediting period	−2	0	Project area is not a protected area.
g)	Mitigation: Where disputes over land tenure, ownership or access/use rights exist, documented evidence is provided that projects have implemented activities to resolve the disputes or clarify overlapping claims	−2	0	Not applicable.
	Total for land tenure and resource access/impacts		2	
	B2. Community engagement risks			
a-b)	Less than 20 percent of households living within 20 km of the project boundary outside the project area, and who are reliant on the project area, have been consulted	5	5	
c)	Mitigation: The project generates net positive impacts on the social and economic well-being of the local communities who derive livelihoods from the project area	−5	−5	Reducing logging intensity improves the availability of non-wood forest products, especially those originating from commercial species.
	Total for community engagement		0	
	B3. Political risks			

	Risk	Value	Score	Explanation
a-e)	Governance score of -0.79 to less than -0.32	0–6	4	The mean of Governance Scores of Gabon across the six indicators of the World Bank Institute's Worldwide Governance Indicators, averaged over 2008–2012 equals -0.555.
f)	Mitigation: Country is implementing REDD+ Readiness or other activities, as set out in Section 2.3.3.	-2	-2	Gabon has an established national PEFC standards body.
		Total for political risks	2	
		Total for external risks	4	(≤ 20 for eligibility)
C. Natural risks				
	Fire		0	
	Pest and disease outbreaks		0	
	Extreme weather		0	
	Geological risk		0	
	Other natural risk		0	
		Total for natural risks	0	(≤ 35 for eligibility)
		Overall risk rating	22	(≤ 60 for eligibility)

3 Species specific parameters and volume equations

Species nomenclature follows the African Plant Database version 3.3.4 of the Conservatoire et Jardin botaniques de la ville de Genve, Switzerland and South African National Biodiversity Institute, Pretoria, retrieved September 2011 from `http://www.ville-ge.ch/musinfo/bd/cjb/africa/`. For volume equations, dbh is expressed in m and volume is given in m^3. Wood density (w_s) is in g cm^{-3}. The administrative minimum cutting limits (A_s) are in cm.

Group	Name	Latin name	w_s	A_s	Volume equation
1	Okoume	*Aucoumea klaineana*	0.3781	70	$-1.8236D + 10.725D^2$
2	Acajou	*Khaya ivorensis*	0.4305	80	$10.82D^{1.89}$
2	Agba (Tola)	*Prioria balsamifera*	0.4133	80	$11.7D^{2.16}$
2	Andoung 66	*Tetraberlinia polyphylla*	0.5301	70	$9.28D^{2.07}$
2	Andoung Heitz	*Aphanocalyx heitzii*	0.4592	70	$9.28D^{2.07}$
2	Andoung Le Testu	*Bikinia letestui*	0.6	70	$9.28D^{2.07}$
2	Anzem Rouge	*Copaifera religiosa*	0.5231	90	$9.72D^{2.46}$
2	Azobe	*Lophira alata*	0.8972	80	$9.72D^{2.46}$
2	Beli	*Julbernardia pellegriniana*	0.6846	100	$9.28D^{2.07}$
2	Bosse Clair	*Guarea cedrata*	0.5074	80	$9.72D^{2.46}$
2	Bosse Fonce	*Guarea thompsonii*	0.5596	80	$9.72D^{2.46}$
2	Dabema	*Piptadeniastrum africanum*	0.6004	70	$9.72D^{2.46}$
2	Dibetou	*Lovoa trichilioides*	0.4554	80	$0.48 + 10.2D^2$
2	Doussie Bella	*Afzelia sp.* (except *bipindensis*, *pachyloba*)	0.6888	80	$9.72D^{2.46}$
2	Doussie Blanc	*Afzelia bipindensis*	0.706	80	$0.6 + 10.8D^2$
2	Doussie Pachyloba	*Afzelia pachyloba*	0.6725	80	$9.72D^{2.46}$
2	Ebiara	*Berlinia bracteosa*	0.6071	70	$9.28D^{2.07}$
2	Eyoum	*Dialium sp.*	0.83	70	$9.72D^{2.46}$
2	Gheombi	*Sindoropsis letestui*	0.6449	70	$9.28D^{2.07}$
2	Gombe	*Didelotia letouzeyi*	0.501	70	$9.28D^{2.07}$
2	Igaganga	*Dacryodes igaganga*	0.5388	70	$9.72D^{2.46}$
2	Iroko	*Milicia excelsa*	0.5795	100	$1.05 + 10.08D^2$
2	Izombe	*Testulea gabonensis*	0.6465	80	$9.72D^{2.46}$

Group	Name	Latin name	w_s	A_s	Volume equation
2	Kevazingo (Bubinga)	*Guibourtia tessmannii*	0.7641	100	$1.05 + 10.08D^2$
2	Kosipo	*Entandrophragma candollei*	0.5959	110	$10.82D^{1.89}$
2	Movingui	*Distemonanthus benthamianus*	0.6014	80	$0.04 + 9.07D^2$
2	Okan	*Cylicodiscus gabunensis*	0.7998	70	$9.72D^{2.46}$
2	Olon	*Zanthoxylum heitzii*	0.4317	70	$9.72D^{2.46}$
2	Omvong	*Dialium pachyphyllum*	0.9225	70	$9.72D^{2.46}$
2	Ossabel	*Dacryodes normandii*	0.5152	60	$9.2D^{1.9}$
2	Ovengkol	*Guibourtia ehie*	0.7096	60	$9.72D^{2.46}$
2	Padouk	*Pterocarpus soyauxii*	0.6402	80	$9.72D^{2.46}$
2	Pau Rosa	*Bobgunnia fistuloides*	0.6	70	$9.72D^{2.46}$
2	Sapelli	*Entandrophragma cylindricum*	0.5782	110	$10.82D^{1.89}$
2	Sipo	*Entandrophragma utile*	0.5436	110	$10.82D^{1.89}$
2	Tali	*Erythrophleum ivorense*	0.7747	70	$9.72D^{2.46}$
2	Tiama Blanc	*Entandrophragma angolense*	0.4702	90	$10.82D^{1.89}$
2	Tiama Noir=Acuminata	*Entandrophragma congoense*	0.4736	80	$10.82D^{1.89}$
2	Wenge	*Millettia laurentii*	0.7469	70	$9.72D^{2.46}$
3	Andoung Durand	*Bikinia durandii*	0.6	70	$9.28D^{2.07}$
3	Andoung Inc	*Bikinia sp.* (except *letestui*, *durandii*, *coriacea*)	0.6	70	$9.28D^{2.07}$
3	Andoung Morel	*Bikinia coriacea*	0.6	70	$9.28D^{2.07}$
3	Anzem Noir, Andem-E.	*Copaifera mildbraedii*	0.6589	90	$9.28D^{2.07}$
4	Aiele	*Canarium schweinfurthii*	0.4121	80	$9.72D^{2.46}$
4	Alen	*Detarium macrocarpum*	0.7026	70	$9.72D^{2.46}$
4	Alep	*Desbordesia glaucescens*	0.9209	70	$9.72D^{2.46}$
4	Alone	*Bombax brevicuspe*	0.4008	70	$9.72D^{2.46}$
4	Alumbi	*Julbernardia seretii*	0.6888	70	$9.72D^{2.46}$
4	Angoa	*Erismadelphus exsul*	0.5841	70	$9.72D^{2.46}$
4	Angueuk	*Ongokea gore*	0.7471	70	$9.72D^{2.46}$
4	Bilinga	*Nauclea diderrichii*	0.67	80	$9.72D^{2.46}$
4	Ebiara-Minkoul	*Berlinia confusa*	0.602	60	$9.28D^{2.07}$
4	Ekaba, Ekop	*Tetraberlinia bifoliolata*	0.4968	70	$9.72D^{2.46}$
4	Faro	*Daniellia sp.*	0.448	70	$9.72D^{2.46}$
4	Fromager	*Ceiba pentandra*	0.2838	70	$9.72D^{2.46}$
4	Ilomba	*Pycnanthus angolensis*	0.3975	70	$11.24D^{1.96}$
4	Kotibe	*Nesogordonia leplaei*	0.7174	70	$9.72D^{2.46}$
4	Limbali	*Gilbertiodendron dewevrei*	0.707	70	$10.34D^{2.22}$
4	Longhi Abam	*Chrysophyllum lacourtianum*	0.6305	70	$9.72D^{2.46}$
4	Longhi Bouk	*Chrysophyllum boukokonse*	0.6458	70	$9.72D^{2.46}$
4	Longhi Mbemame	*Chrysophyllum africanum*	0.63	70	$9.72D^{2.46}$
4	Longhi Perpulchra	*Chrysophyllum perpulchrum*	0.7052	70	$9.28D^{2.07}$
4	Longhi Subnuda	*Chrysophyllum subnudum*	0.6402	70	$9.72D^{2.46}$
4	Mekogho (Faux-Tali)	*Pachyelasma tessmannii*	0.7381	70	$9.72D^{2.46}$
4	Mukulungu	*Autranella congolensis*	0.78	90	$9.72D^{2.46}$
4	Niove	*Staudtia kamerunensis*	0.7886	60	$11.24D^{1.96}$
4	Onzabili	*Antrocaryon klaineanum*	0.5231	60	$9.28D^{2.07}$
4	Owui	*Hexalobus crispiflorus*	0.5016	70	$9.72D^{2.46}$
4	Tchitola	*Prioria oxyphylla*	0.5519	70	$9.72D^{2.46}$
5	Abeum	*Gilbertiodendron klainei*	0.6587	70	$9.72D^{2.46}$

Group	Name	Latin name	w_s	A_s	Volume equation
5	Agnuhe	*Pentadesma butyracea*	0.7784	70	$9.72D^{2.46}$
5	Ako	*Antiaris toxicaria*	0.3728	70	$9.72D^{2.46}$
5	Bodioa	*Anopyxis klaineana*	0.7785	70	$9.72D^{2.46}$
5	Bombax	*Bombax buonopozense*	0.3229	70	$9.72D^{2.46}$
5	Coula	*Coula edulis*	0.9135	70	$9.72D^{2.46}$
5	Dacryodes	*Dacryodes klaineana*	0.7177	70	$9.72D^{2.46}$
5	Diania	*Celtis tessmannii*	0.6964	70	$9.72D^{2.46}$
5	Divida	*Scorodophloeus zenkeri*	0.7428	70	$9.72D^{2.46}$
5	Edji	*Amphimas ferrugineus*	0.667	70	$9.72D^{2.46}$
5	Ekoulebang	*Parinari glabra*	0.9127	70	$9.72D^{2.46}$
5	Ekoune	*Coelocaryon preussii*	0.5019	70	$11.24D^{1.96}$
5	Emien	*Alstonia sp.*	0.3874	70	$9.72D^{2.46}$
5	Essang	*Parkia bicolor*	0.4304	70	$9.72D^{2.46}$
5	Essang-Eli	*Parinari sp.*	0.69	70	$9.72D^{2.46}$
5	Essessang	*Ricinodendron heudelotii*	0.2069	70	$9.72D^{2.46}$
5	Essia	*Petersianthus macrocarpus*	0.6905	70	$9.72D^{2.46}$
5	Essong	*Irvingia robur*	0.8026	70	$9.72D^{2.46}$
5	Etom	*Syzygium staudtii*	0.6328	70	$9.72D^{2.46}$
5	Eveuss	*Klainedoxa gabonensis*	0.9241	70	$9.72D^{2.46}$
5	Evino	*Vitex sp.*	0.52	70	$9.72D^{2.46}$
5	Faux Padouk	*Pterocarpus tessmannii*	0.5844	70	$9.72D^{2.46}$
5	Gambeya	*Chrysophyllum sp.* (except *lacourtianum*, *boukokonse*, *africanum*, *perpulchrum*, *subnudum*)	0.6458	70	$9.72D^{2.46}$
5	Kong-Afane	*Letestua durissima*	0.9722	70	$9.72D^{2.46}$
5	Landa	*Erythroxylum mannii*	0.5519	70	$9.72D^{2.46}$
5	Lannea	*Lannea welwitschii*	0.4303	70	$9.72D^{2.46}$
5	Manil	*Symphonia globulifera*	0.595	50	$9.72D^{2.46}$
5	Mbanegue	*Gilletiodendron pierreanum*	0.8904	70	$9.72D^{2.46}$
5	Mubala	*Pentaclethra macrophylla*	0.8241	70	$9.72D^{2.46}$
5	Mvana	*Hylodendron gabunense*	0.7869	70	$9.72D^{2.46}$
5	Ngaba	*Librevillea klainei*	0.9055	70	$9.72D^{2.46}$
5	Ngang Pf	*Hymenostegia pellegrinii*	0.835	70	$9.72D^{2.46}$
5	Ngong Mebame	*Funtumia africana*	0.4156	70	$9.72D^{2.46}$
5	Nieuk	*Fillaeopsis discophora*	0.4838	70	$9.72D^{2.46}$
5	Nka	*Pteleopsis hylodendron*	0.6807	70	$9.72D^{2.46}$
5	Nkagha	*Tessmannia africana*	0.8242	70	$9.72D^{2.46}$
5	Ntana	*Marquesia excelsa*	0.7577	70	$9.72D^{2.46}$
5	Oboto	*Mammea usambarensis*	0.6465	70	$9.72D^{2.46}$
5	Oddonio	*Oddoniodendron sp.*	0.9375	70	$9.72D^{2.46}$
5	Ohia	*Celtis mildbraedii*	0.6027	70	$9.72D^{2.46}$
5	Okolangouma	*Lecomtedoxa klaineana*	0.8621	70	$9.72D^{2.46}$
5	Olene	*Irvingia grandifolia*	0.8006	70	$9.72D^{2.46}$
5	Olonvogo	*Zanthoxylum gilletii*	0.6888	70	$9.72D^{2.46}$
5	Onzan	*Odyendyea gabonensis*	0.6	70	$9.72D^{2.46}$
5	Ossang-Eli	*Parinari hypochrysea*	0.69	70	$9.72D^{2.46}$
5	Ossimiale	*Newtonia leucocarpa*	0.5971	70	$9.72D^{2.46}$
5	Rikio	*Uapaca sp.*	0.645	70	$9.72D^{2.46}$
5	Sene	*Albizia adianthifolia*	0.495	70	$9.72D^{2.46}$

Group	Name	Latin name	w_s	A_s	Volume equation
5	Sorro	*Scyphocephalium mannii*	0.5101	60	$9.72D^{2.46}$
5	Stemeno	*Stemonocoleus micranthus*	0.5809	70	$9.72D^{2.46}$
6	Acioa	*Dactyladenia sp.*	0.6	70	$9.72D^{2.46}$
6	Adjouaba	*Dacryodes klaineana*	0.7177	70	$9.72D^{2.46}$
6	Adzacon	*Lecomtedoxa nogo*	0.8621	70	$9.72D^{2.46}$
6	Adzacon-Aboga	*Manilkara fouilloyana*	0.861	70	$9.72D^{2.46}$
6	Adzem	*Psilanthus mannii*	0.6	70	$9.72D^{2.46}$
6	Afane	*Panda oleosa*	0.5652	70	$9.72D^{2.46}$
6	Afatouk	*Maranthes gabunensis*	0.8292	70	$9.72D^{2.46}$
6	Afina	*Strombosia pustulata*	0.8421	70	$9.72D^{2.46}$
6	Afoupeli	*Hypodaphnis zenkeri*	0.6	70	$9.72D^{2.46}$
6	Ahinebe	*Anthocleista sp.*	0.5266	70	$9.72D^{2.46}$
6	Akak	*Duboscia macrocarpa*	0.6	70	$9.72D^{2.46}$
6	Ake	*Pterygota bequaertii*	0.5243	70	$9.72D^{2.46}$
6	Akeul	*Pausinystalia macroceras*	0.5876	70	$9.72D^{2.46}$
6	Akok	*Baphia sp.*	0.5678	70	$9.72D^{2.46}$
6	Akol	*Ficus exasperata*	0.3444	70	$9.72D^{2.46}$
6	Akom	*Beilschmiedia fulva*	0.5732	70	$9.72D^{2.46}$
6	Akot	*Drypetes gossweileri*	0.6629	70	$9.72D^{2.46}$
6	Alane Beku	*Klaineanthus gaboniae*	0.6	70	$9.72D^{2.46}$
6	Allen-Ocpo	*Dracaena sp.*	0.4166	70	$9.72D^{2.46}$
6	Allophyllus	*Allophylus sp.*	0.53	70	$9.72D^{2.46}$
6	Amvout	*Trichoscypha oddonii*	0.6285	70	$9.72D^{2.46}$
6	Andong	*Strephonema sericeum*	0.6291	70	$9.72D^{2.46}$
6	Angylocalyx	*Angylocalyx sp.*	0.6	70	$9.72D^{2.46}$
6	Anthonotha	*Anthonotha sp.* (except *fragrans*)	0.8241	70	$9.72D^{2.46}$
6	Antidesma	*Antidesma sp.*	0.68	70	$9.72D^{2.46}$
6	Anzilim	*Eurypetalum sp.*	0.6	70	$9.72D^{2.46}$
6	Aphanocalyx	*Aphanocalyx sp.* (except *heitzii*)	0.4592	70	$9.72D^{2.46}$
6	Arbre De La Passion	*Paropsia grewioides*	0.675	70	$9.72D^{2.46}$
6	Assas	*Macaranga sp.*	0.336	70	$9.72D^{2.46}$
6	Atangatier	*Dacryodes edulis*	0.5162	70	$9.72D^{2.46}$
6	Atieghe	*Discoglypremna caloneura*	0.3424	70	$9.72D^{2.46}$
6	Atom	*Dacryodes macrophylla*	0.5533	70	$9.72D^{2.46}$
6	Atsui	*Vismia rubescens*	0.489	70	$9.72D^{2.46}$
6	Aubrevillea	*Aubrevillea sp.*	0.6	70	$9.72D^{2.46}$
6	Avie	*Memecylon sp.*	0.785	70	$9.72D^{2.46}$
6	Avom	*Cleistopholis patens*	0.3556	70	$9.72D^{2.46}$
6	Baikia	*Baikiaea sp.*	0.7545	70	$9.72D^{2.46}$
6	Balanites	*Balanites wilsoniana*	0.6629	70	$9.72D^{2.46}$
6	Balonga (Otounga Gf)	*Balonga buchholzii*	0.6	70	$9.72D^{2.46}$
6	Beniaman	*Tetraberlinia moreliana*	0.5301	70	$9.72D^{2.46}$
6	Berlinia	*Berlinia sp.* (except *bracteosa*, *congolensis*)	0.6139	70	$9.72D^{2.46}$
6	Blighia	*Blighia welwitschii*	0.7819	70	$9.72D^{2.46}$
6	Bong	*Zanthoxylum tessmannii*	0.6003	70	$9.72D^{2.46}$
6	Brazzeia	*Brazzeia sp.*	0.6	70	$9.72D^{2.46}$
6	Camptostylus	*Oncoba mannii*	0.58	70	$9.72D^{2.46}$
6	Canthium	*Canthium sp.*	0.7224	70	$9.72D^{2.46}$

Group	Name	Latin name	w_s	A_s	Volume equation
6	Cassipourea	*Cassipourea sp.*	0.6197	70	$9.72D^{2.46}$
6	Chytranthus	*Chytranthus sp.*	0.6	70	$9.72D^{2.46}$
6	Claoxylon	*Claoxylon sp.*	0.355	70	$9.72D^{2.46}$
6	Coffea	*Coffea sp.*	0.6328	70	$9.72D^{2.46}$
6	Cola	*Cola sp.*	0.5231	70	$9.72D^{2.46}$
6	Conceveiba	*Conceveiba macrostachys*	0.504	70	$9.72D^{2.46}$
6	Crabwood	*Carapa procera*	0.5684	70	$9.72D^{2.46}$
6	Crossopteryx	*Crossopteryx sp.*	0.7017	70	$9.72D^{2.46}$
6	Crudia	*Crudia sp.*	0.8	70	$9.72D^{2.46}$
6	Cryptosepalum	*Cryptosepalum sp.*	0.7601	70	$9.72D^{2.46}$
6	Cuviera	*Cuviera sp.*	0.6	70	$9.72D^{2.46}$
6	Dibeum	*Gilbertiodendron unijugum*	0.6629	70	$9.72D^{2.46}$
6	Domele	*Bertiera sp.*	0.6	70	$9.72D^{2.46}$
6	Drypetes	*Drypetes sp.* (except *gossweileri*)	0.7146	70	$9.72D^{2.46}$
6	Duvigne	*Duvigneaudia inopinata*	0.6	70	$9.72D^{2.46}$
6	Ebam	*Picralima nitida*	0.7749	70	$9.72D^{2.46}$
6	Ebebeng	*Margaritaria discoidea*	0.7195	70	$9.72D^{2.46}$
6	Ebene	*Diospyros sp.* (except *crassiflora*)	0.7	70	$9.28D^{2.07}$
6	Ebene Noir	*Diospyros crassiflora*	0.8261	70	$9.28D^{2.07}$
6	Ebo	*Santiria trimera*	0.5554	70	$9.72D^{2.46}$
6	Eboboku	*Scaphopetalum sp.*	0.6	70	$9.72D^{2.46}$
6	Ebom	*Anonidium mannii*	0.2913	70	$9.72D^{2.46}$
6	Ebom Rouge	unknown	0.6	70	$9.72D^{2.46}$
6	Edzip	*Strombosia grandifolia*	0.8164	70	$9.72D^{2.46}$
6	Efot	*Magnistipula tessmannii*	0.6	70	$9.72D^{2.46}$
6	Egipt	*Strombosiopsis tetrandra*	0.6629	70	$9.72D^{2.46}$
6	Ekaku	*Thomandersia sp.*	0.6	70	$9.72D^{2.46}$
6	Ekat	*Neochevalierodendron stephanii*	0.6	70	$9.72D^{2.46}$
6	Ekoba	*Diogoa zenkeri*	0.6955	70	$9.72D^{2.46}$
6	Emvi	*Homalium longistylum*	0.73	70	$9.72D^{2.46}$
6	Endodesmia	*Endodesmia sp.*	0.6951	70	$9.72D^{2.46}$
6	Endone	*Pausinystalia johimbe*	0.5876	70	$9.72D^{2.46}$
6	Engokom	*Barteria fistulosa*	0.6	70	$9.72D^{2.46}$
6	Engomegoma	*Engomegoma gordonii*	0.6	70	$9.72D^{2.46}$
6	Engona	*Pentaclethra eetveldeana*	0.6629	70	$9.72D^{2.46}$
6	Engong	*Trichoscypha engong*	0.6285	70	$9.72D^{2.46}$
6	Erythrina	*Erythrina sp.*	0.264	70	$9.72D^{2.46}$
6	Esoma	*Rauvolfia caffra*	0.4525	70	$9.72D^{2.46}$
6	Essoula	*Plagiostyles africana*	0.7381	70	$9.72D^{2.46}$
6	Etou	*Treculia africana*	0.6	70	$9.72D^{2.46}$
6	Etua	*Tabernaemontana crassa*	0.5504	70	$9.72D^{2.46}$
6	Evegna	*Microdesmis puberula*	0.6	70	$9.72D^{2.46}$
6	Evegveu	*Irvingia excelsa*	0.8026	70	$9.72D^{2.46}$
6	Evong-Evong	*Spathodea campanulata*	0.2712	70	$9.72D^{2.46}$
6	Ewoleghe	*Bridelia sp.*	0.5328	70	$9.72D^{2.46}$
6	Ezelfou	*Sterculia tragacantha*	0.43	70	$9.72D^{2.46}$
6	Feup	*Isolona hexaloba*	0.6	70	$9.72D^{2.46}$

Group	Name	Latin name	w_s	A_s	Volume equation
6	Ficus Arbre	*Ficus vogeliana*	0.4004	70	$9.72D^{2.46}$
6	Ficus Etrangleur	*Ficus sp.* (except *vogeliana*, *mucuso*, *exasperata*)	0.4004	70	$9.72D^{2.46}$
6	Ganophyllum	*Ganophyllum giganteum*	0.698	70	$9.72D^{2.46}$
6	Garcinia	*Garcinia sp.*	0.7293	70	$9.72D^{2.46}$
6	Gardenia	*Gardenia imperialis*	0.6665	70	$9.72D^{2.46}$
6	Ghekoa	*Vepris soyauxii*	0.6591	70	$9.72D^{2.46}$
6	Gilbertiodendron	*Gilbertiodendron sp.*	0.6629	70	$9.72D^{2.46}$
6	Grewia	*Grewia sp.*	0.57	70	$9.72D^{2.46}$
6	Guarea	*Guarea sp.* (except *cedrata*, *thompsonii*)	0.545	70	$9.72D^{2.46}$
6	Homalium	*Homalium sp.* (except *longistylum*, *letestui*)	0.73	70	$9.72D^{2.46}$
6	Hymeno	*Hymenostegia ngounyensis*	0.8296	70	$9.72D^{2.46}$
6	Inconnue	unknown	0.6	70	$9.72D^{2.46}$
6	Isolona	*Isolona hexaloba*	0.6	70	$9.72D^{2.46}$
6	Ka	*Dichostemma glaucescens*	0.6	70	$9.72D^{2.46}$
6	Kanguele	*Maesopsis eminii*	0.3901	70	$9.72D^{2.46}$
6	Kaoue	*Stachyothyrsus staudtii*	0.645	70	$9.72D^{2.46}$
6	Kobahia	*Christiana africana*	0.6	70	$9.72D^{2.46}$
6	Lebonda	*Trichilia tessmannii*	0.64	70	$9.72D^{2.46}$
6	Lembesse	*Centroplacus glaucinus*	0.6	70	$9.72D^{2.46}$
6	Lepoute	*Maranthes aubrevillei*	0.8292	70	$9.72D^{2.46}$
6	Maranthes	*Maranthes sp.* (except *glabra*, *gabunensis*, *chrysophylla*)	0.8292	70	$9.72D^{2.46}$
6	Mareya	*Mareya sp.*	0.6	70	$9.72D^{2.46}$
6	Mebimengone	*Omphalocarpum elatum*	0.5504	70	$9.72D^{2.46}$
6	Med	*Cyrtogonone argentea*	0.6	70	$9.72D^{2.46}$
6	Medzime Koghe	*Psychotria sp.*	0.52	70	$9.72D^{2.46}$
6	Mengo	*Aoranthe cladantha*	0.8064	70	$9.72D^{2.46}$
6	Mfol	*Annickia chlorantha*	0.4462	70	$9.72D^{2.46}$
6	Miama	*Calpocalyx heitzii*	0.7168	70	$9.72D^{2.46}$
6	Miamengone	*Oncoba welwitschii*	0.58	70	$9.72D^{2.46}$
6	Millettia	*Millettia sp.* (except *laurentii*)	0.7381	70	$9.72D^{2.46}$
6	Mississe	*Calpocalyx sp.* (except *heitzii*)	0.7135	70	$9.72D^{2.46}$
6	Mondjadi	*Crateranthus talbotii*	0.6	70	$9.72D^{2.46}$
6	Morinda	*Morinda lucida*	0.54	70	$9.72D^{2.46}$
6	Mugondi	*Eriocoelum sp.*	0.5231	70	$9.72D^{2.46}$
6	Mvezork	*Homalium letestui*	0.7084	70	$9.72D^{2.46}$
6	Mvouma	*Xylopia quintasii*	0.75	70	$9.72D^{2.46}$
6	Napoleona	*Napoleona sp.*	0.6	70	$9.72D^{2.46}$
6	Ndande	*Xylopia phloiodora*	0.5909	70	$9.72D^{2.46}$
6	Ndong-Eli	*Xylopia hypolampra*	0.6629	70	$9.72D^{2.46}$
6	Ngang Gf	*Hymenostegia klainei*	0.8296	70	$9.72D^{2.46}$
6	Ngang Inconnu	*Hymenostegia sp.* (except *pellegrinii*, *klainei*, *ngounyensis*)	0.8296	70	$9.72D^{2.46}$
6	Ngeul Fv	*Croton sylvaticus*	0.5231	70	$9.72D^{2.46}$

Group	Name	Latin name	w_s	A_s	Volume equation
6	Ngorangorane	*Oncoba glauca*	0.58	70	$9.72D^{2.46}$
6	Ngueul Fb	*Croton mayumbensis*	0.5231	70	$9.72D^{2.46}$
6	Nkonengu	*Beilschmiedia pierreana*	0.5732	70	$9.72D^{2.46}$
6	Nkouarsa	*Tetrapleura tetraptera*	0.5312	70	$9.72D^{2.46}$
6	Nsa	*Maprounea membranacea*	0.57	70	$9.72D^{2.46}$
6	Nsire	unknown	0.6	70	$9.72D^{2.46}$
6	Ntom	*Duguetia staudtii*	0.6332	70	$9.72D^{2.46}$
6	Ntoma Biliba	*Sarcocephalus pobeguinii*, *Nauclea vanderguchtii*	0.4976	70	$9.72D^{2.46}$
6	Ntona	*Xylopia pynaertii*	0.5909	70	$9.72D^{2.46}$
6	Ntsua	*Xylopia rubescens*, *Xylopia staudtii*	0.5909	70	$9.72D^{2.46}$
6	Nzang	*Synsepalum afzelii*	0.8153	70	$9.72D^{2.46}$
6	Nzim Soreu	*Anisophyllea myriosticta*	0.72	70	$9.72D^{2.46}$
6	Oboba	*Myrianthus arboreus*	0.4496	70	$9.72D^{2.46}$
6	Ochtocosmus	*Phyllocosmus sp.*	0.78	70	$9.72D^{2.46}$
6	Oduma	*Prioria joveri*	0.42	70	$9.72D^{2.46}$
6	Odzicouna	*Scytopetalum klaineanum*	0.6155	70	$9.72D^{2.46}$
6	Ofira	*Aubrevillea platycarpa*	0.6	70	$9.72D^{2.46}$
6	Ofoss	*Pseudospondias microcarpa*	0.6	70	$9.72D^{2.46}$
6	Okala	*Xylopia aethiopica*	0.4422	70	$9.72D^{2.46}$
6	Oncoba	*Oncoba sp.* (except *glauca*, *welwitschii*)	0.58	70	$9.72D^{2.46}$
6	Onzem	*Anthonotha fragrans*	0.5291	70	$9.72D^{2.46}$
6	Otounga	*Polyalthia suaveolens*	0.6951	70	$9.72D^{2.46}$
6	Ovita	*Afrostyrax sp.*	0.6	70	$9.72D^{2.46}$
6	Ovok	*Cleistopholis glauca*	0.3045	70	$9.72D^{2.46}$
6	Oyem	*Brenania brieyi*	0.6	70	$9.72D^{2.46}$
6	Oyem Tsue	*Rauvolfia vomitoria*	0.4698	70	$9.72D^{2.46}$
6	Oyop	*Chrysophyllum sp.*	0.6458	70	$9.72D^{2.46}$
6	Palmier A Huile	*Elaeis guineensis*	0.6	70	$9.72D^{2.46}$
6	Parasolier	*Musanga cecropioides*	0.2289	70	$9.72D^{2.46}$
6	Passa	*Heisteria parvifolia*	0.705	70	$9.72D^{2.46}$
6	Pierrodendron	*Quassia grandifolia*	0.331	70	$9.72D^{2.46}$
6	Plagiosiphon	*Plagiosiphon sp.*	0.6	70	$9.72D^{2.46}$
6	Protomeg	*Protomegabaria macrophylla*	0.602	70	$9.72D^{2.46}$
6	Rhabdophyllum	*Rhabdophyllum sp.*	0.6	70	$9.72D^{2.46}$
6	Rinorea	*Rinorea sp.*	0.682	70	$9.72D^{2.46}$
6	Rothmania	*Rothmannia sp.*	0.6414	70	$9.72D^{2.46}$
6	Sabifout	*Maesobotrya sp.*	0.6	70	$9.72D^{2.46}$
6	Samanea	*Samanea leptophylla*	0.6	70	$9.72D^{2.46}$
6	Sangoma	*Allanblackia parviflora*	0.5456	70	$9.72D^{2.46}$
6	Sapium	*Sclerocroton sp.*, *Shirakiopsis sp.*	0.6	70	$9.72D^{2.46}$
6	Scottellia	*Scottellia sp.*	0.5485	70	$9.72D^{2.46}$
6	Set	*Cryptosepalum congolanum*	0.7601	70	$9.72D^{2.46}$
6	Sorindeia	*Sorindeia sp.*	0.56	70	$9.72D^{2.46}$
6	Strychnos	*Strychnos sp.*	0.7017	70	$9.72D^{2.46}$
6	Synsepalum	*Synsepalum sp.*	0.6776	70	$9.72D^{2.46}$
6	Tol	*Ficus mucuso*	0.4091	70	$9.72D^{2.46}$

Group	Name	Latin name	w_s	A_s	Volume equation
6	Tricalysia	*Tricalysia sp.*	0.1	70	$9.72D^{2.46}$
6	Trichilia	*Trichilia sp.* (except *tessmannii*)	0.64	70	$9.72D^{2.46}$
6	Trichoscypha	*Trichoscypha sp.* (except *oddonii*, *acuminata*, *engong*)	0.6285	70	$9.72D^{2.46}$
6	Uvariastrum	*Uvariastrum sp.*	0.6	70	$9.72D^{2.46}$
6	Vangueriopsis	*Vangueriella sp.*	0.6	70	$9.72D^{2.46}$
6	Warneckea	*Warneckea sp.*	0.6	70	$9.72D^{2.46}$
6	Xylopia	*Xylopia sp.* (except *aethiopica*, *hypolampra*, *staudtii*, *quintasii*, *rubescens*)	0.5909	70	$9.72D^{2.46}$
7	Andok	*Irvingia gabonensis*	0.7902	70	$9.72D^{2.46}$
7	Bahia (Abura)	*Hallea ledermannii*	0.4685	70	$9.72D^{2.46}$
7	Douka	*Tieghemella africana*	0.6201	100	$0.72 + 11.32D^2$
7	Moabi	*Baillonella toxisperma*	0.7259	100	$11.59D^{1.94}$
7	Ovoga (Afo)	*Poga oleosa*	0.3657	60	$9.72D^{2.46}$
7	Ozigo	*Dacryodes buettneri*	0.5001	80	$9.2D^{1.9}$

References

TEREA, 2013. Concession REDD+ certifiée FSC du Haut-Nyong : augmentation des DMA et certification FSC. Rapport du projet FORAFAMA, Cameroun.

Verified Carbon Standard, 2012. AFOLU non-permanence risk tool, version 3.2. Procedural document, VCS Association, Washington, DC, USA.
