# Supplementary figures and images for: Opportunity costs of carbon sequestration in a forest concession in central Africa
Source: Carbon Balance Manag. 2014 Jul 3;9:4. doi: 10.1186/s13021-014-0004-3 (PMC4637000; doi:10.1186/s13021-014-0004-3)

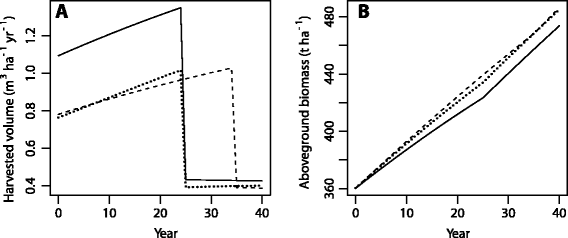

Supplement: Supplementary file 4 — Authors’ original file for figure 1 [file 13021_2014_4_MOESM4_ESM.gif]

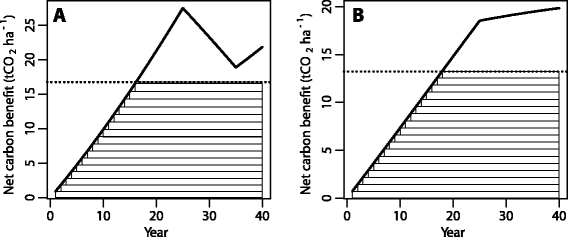

Supplement: Supplementary file 5 — Authors’ original file for figure 2 [file 13021_2014_4_MOESM5_ESM.gif]

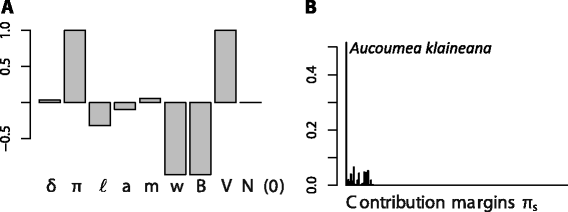

Supplement: Supplementary file 6 — Authors’ original file for figure 3 [file 13021_2014_4_MOESM6_ESM.gif]
